# Frequency Dependence of Receiving Sensitivity of Ultrasonic Transducers and Acoustic Emission Sensors

**DOI:** 10.3390/s18113861

**Published:** 2018-11-09

**Authors:** Kanji Ono

**Affiliations:** Department of Materials Science and Engineering, University of California, Los Angeles (UCLA), Los Angeles, CA 90095, USA; ono@ucla.edu; Tel.: +1-310-825-5534

**Keywords:** ultrasonic transducers, acoustic emission sensors, receiving sensitivity, low frequency characteristics, sinewave excitation, impulse method, open-circuit sensitivity, input impedance, frequency independent sensitivity, damped harmonic oscillator, minimum pulse duration

## Abstract

Receiving displacement sensitivities (Rx) of ultrasonic transducers and acoustic emission (AE) sensors are evaluated using sinewave packet excitation method and compared to the corresponding data from pulse excitation method with a particular emphasis on low frequency behavior below 20 kHz, down to 10 Hz. Both methods rely on the determination of transmitter displacement characteristics using a laser interferometric method. Results obtained by two calibration methods are in good agreement, with average spectral differences below 1 dB, indicating that the two calibration methods yield identical receiving sensitivities. At low test frequencies, effects of attenuation increase substantially due to increasing sensor impedance and Rx requires correction in order to evaluate the inherent sensitivity of a sensor, or open-circuit sensitivity. This can differ by more than 20 dB from results that used common preamplifiers with ~10 kΩ input impedance, leading to apparent velocity response below 100 kHz for typical AE sensors. Damped broadband sensors and ultrasonic transducers exhibit inherent velocity response (Type 1) below their main resonance frequency. In sensors with under-damped resonance, a steep sensitivity decrease occurs showing frequency dependence of f^2^~f^5^ (Type 2), while mass-loaded sensors exhibit flat displacement response (Type 0). Such behaviors originate from sensor characteristics that can best be described by the damped harmonic oscillator model. This model accounts for the three typical behaviors. At low frequencies, typically below 1 kHz, receiving sensitivity exhibits another Type 0 behavior of frequency independent Rx. Seven of 12 sensors showed this flat region, while three more appear to approach the Type 0 region. This appears to originate from the quasi-static piezoelectric response of a sensing element. In using impulse method, a minimum pulse duration is necessary to obtain spectral fidelity at low frequencies and an approximate rule is given. Various factors for sensitivity improvement are also discussed.

## 1. Introduction

### 1.1. Background of Detector Calibration

**Figure 1:**
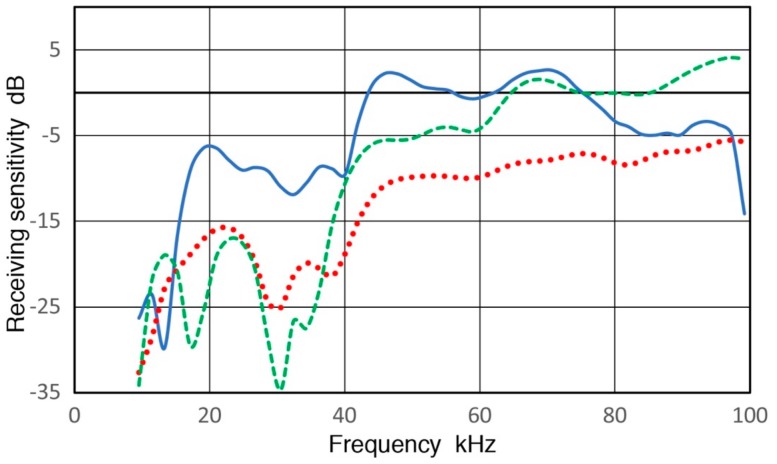
Receiving displacement sensitivities of three low frequency AE sensors. Blue curve: PAC R.45, Red dot: PAC R3a, Green dash: PAC R6a. Sensitivity in dB in reference to 1 V/nm. Input impedance = 10 kΩ.

**Figure 2:**
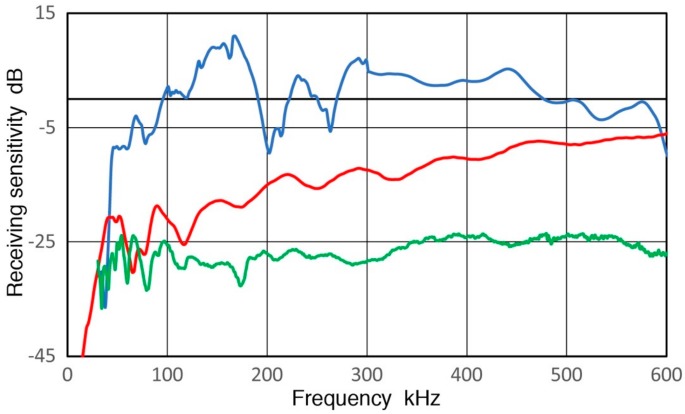
Receiving displacement sensitivities of PAC R15a (blue curve), Olympus V103 (red) and KRN BB-PCP (green). Sensitivity in dB in reference to 1 V/nm. Input impedance = 10 kΩ.

In ultrasonic testing (UT) and acoustic emission (AE) testing, transducers and sensors play key roles in detecting weak signals that arrive with various noise. Detected signals are affected by these detectors, thus having their characteristics is important, especially for system modeling of test procedures [1]. Yet, clear guidelines for the calibration procedures have not been available unlike the case of vibration and shock transducers [2]. Recent studies using pulse excitation and laser interferometry have characterized the transmission and receiving sensitivities of UT transducers and AE sensors [3,4]. This approach is a variation of ISO16063-11 [2] by identifying the transmission behavior of UT transducers and using it for receiving sensitivity (Rx) determination. Some of the results have been verified by tests using a laser vibrometer [5,6]. The studies [3,4] resulted in the frequency dependence of Rx of more than a dozen UT/AE detectors. Three representative Rx spectra are shown in Figure 1 for damped UT transducers (Group D for damping with medium sensitivity levels and a broad peak sensitivity), resonant AE sensors (Group R for resonance having high sensitivity with a series of resonance peaks) and broadband AE sensors (Group B for broadband having lower sensitivity with broad and relatively flat sensitivity). An AE sensor in Group D, aimed toward research and specialized applications, has backing material behind the sensing element, represented here by V103 (Olympus NDT Instruments, Waltham, MA, USA). This shows several sensitivity peaks below 300 kHz from radial resonances and an increasing trend. This is a UT transducer of 1 MHz center frequency and its sensitivity peaking at 800–1000 kHz. The construction details of such UT transducers can be found in [7]. For Group R, a sensitivity curve for R15a from Physical Acoustics (PAC, Princeton Junction, NJ, USA) is shown, representing high-sensitivity AE sensors. This type usually has only a piezoelectric disk without any damping and produces a series of resonance peaks and is for general AE testing uses. This spectrum has radial resonance peaks at 140–170 kHz (reaching 11 dB in reference to 1 V/nm), followed by a thickness resonance peak at 290 kHz. Group B is represented by a KRN BB-PCP sensor (KRN Services, Richland, WA, USA). This kind of AE sensor was invented at the National Institute of Standards and Technology (NIST) by Proctor [8], with broadband behavior built-in. This is mostly used for research. The Proctor design uses a small truncated conical sensing element with a large mass as backing. The KRN sensor is a variation of the NIST conical sensor with a compact design, but still produces a broadband response. In all groups, the sensitivity diminished at low frequencies. This aspect was also observed in AE sensors for low frequency applications, as shown in Figure 2. Three AE sensors are PAC R.45, R3a and R6a and their receiving sensitivities decreased sharply below 10–20 kHz. Such a sensitivity drop is a desirable behavior for conventional AE applications. However, this and other frequency dependence characteristics have not been explored and this study aims to elucidate their origins.

Because these impulse methods [3,4] utilize short-pulse excitation of less than a few µs, it is needed to verify the accuracy of low frequency receiving sensitivities where wave periods are much longer than these pulses. Because of concern on this point, the recent sensor studies [3,4] were limited to usual AE frequency range of >20 kHz. While UT and AE applications usually involve short excitation input, some AE signals can be longer, and this question has not previously been addressed. This situation necessitates an extension of examining the frequency response of the UT/AE detectors down to 1 kHz or below. Here, a variation of Method 3 of ISO16063-11 is useful [2]; that is, sinewave excitation is to be used in place of pulse excitation. This should lead to comprehensive understanding of frequency dependence behavior as well as resolving another question of whether resonance effects are measured adequately using impulse excitation.

Lower AE frequency ranges from 0.1 to 10 kHz have been used in geotechnical fields since the 1930s; see Hardy [9] and Manthei et al. [10]. In AE studies of rock stability, a higher range above 10 kHz has become increasingly useful [11]. An AE study of concrete also used below 10 kHz and observed a rapid rise of geophone AE near final fracture is consistent with a mechanism of fracture-induced AE events [12]. However, AE signals under 20 kHz have mostly been associated with secondary AE from pre-existing cracks [13]. In most low-frequency works, accelerometers and geophones with factory calibration were used. However, these are designed to have flat frequency responses and to work with long sensor cables. Thus, the sensitivity was generally low and was inadequate to detect events recorded by AE sensors [11]. Few works of AE sensor calibration are known using vibrators or shakers that are usually employed for low-frequency evaluation. One report for a 78-kHz AE sensor is found listing 1.2 mV/m/s^2^ sensitivity over 0 to 10 kHz [14], but no frequency response was shown. When a frequency of 1 kHz is chosen, its displacement sensitivity corresponds to −88 dB in reference to 1 V/nm, about 100 dB lower than the peak value for R15a (see Figure 1). If this level is common with other AE sensors, there is a keen need for improving AE sensor sensitivities at low frequencies.

A recent review on large-scale geotechnical applications of AE methods [15] has revealed urgent needs to develop calibration standards for AE sensors at 1–100 kHz range. In two AE studies cited, a large-scale monitoring deduced 360,000 AE sources at a deep mine 1000–3000 m below the ground level [16,17]. For these cases, the AE monitoring system utilized an algorithm that automatically analyzed 150 million multi-sensor signal arrivals [18]. In these tests in the mines, frequency range was higher than traditional geotechnical applications that utilized geophones at frequencies below 1 kHz [19]. In 1996, Dunegan [20] reported that a small aperture sensor from Dunegan Engineering Consultants Inc. (DECI), model SE1000HI, showed a flat sensitivity of −17 to −20 dB in reference to 1 V/nm over 1 to 3 kHz, although no details of this calibration were given. He also noted the availability of its NIST-traceable calibration over 7 to 400 kHz. By examining laboratory records kept at the National Institute of Standards and Technology (NIST) at Boulder, CO, Hamstad [21] recently confirmed that NIST conducted the calibration for an SE1000HI by their surface wave set-up. The sensitivity level for SE1000HI is comparable to that of a recent small aperture sensor (KRN Services, BB-PCP) at higher frequencies, as reported in [3]. Recent works [22,23] have also extended the calibration frequency down to 1 kHz for several UT and AE sensors. This method is based on a ball drop and finite element displacement calculation. Geophones and accelerometers have been widely used in seismology and structural monitoring at even lower frequencies and these are calibrated using methods in ISO16063-11 [2,24]. However, vibrators for these tests are not useful at higher frequencies (above 50–100 kHz). Thus, the low-frequency extension of AE sensor calibration study should cover the range of 1 to 100 kHz in a consistent manner with the existing vibration standards. A novel optical design for highly sensitive seismometers has emerged [25] and it will be interesting to find how close general use AE sensors can approach the sensitivity levels of these specialized instruments.

While UT/AE sensors detect dynamic events, piezoelectric sensors are also useful at much lower frequencies, down to near dc. This part is called quasi-static phenomenon and is subjected to active studies [26,27,28,29]. The quasi-static behavior of UT/AE sensors has not been reported, however.

The use of UT transducers in usual pulse-echo inspection has one prominent factor differing from their applications in AE testing. In a UT pulser-receiver, such as Panametrics 5055PR (Olympus NDT), a damping resistor (60 Ω) for high-voltage pulses constitutes a part of a low impedance input circuit. Most AE preamplifiers, on the other hand, have 10~20 kΩ input impedance, since higher sensitivity and longer sensor cables are usually needed in AE testing.

Except for the resonance-induced peaking and flat displacement response due to mass backing, the latter analyzed in detail by Greenspan [30], the frequency response behavior of these sensors and transducers has not been examined comprehensively. The main reason for this lack is the absence of reliable calibration methods until recently [3,4]. Thus, we aim to correlate the observed frequency response to the damped harmonic oscillator (DHO) model [31], which will be used as the foundation. Another theoretical basis is derived from piezoelectricity that predicts displacement response at low frequencies [28].

### 1.2. Study Objectives

In the present study, we rely on the transmission sensitivities of UT transducers, which were previously calibrated using impulse methodology and laser interferometry [3]. This calibration method utilized the same principle described in ISO16063-11 [2]. To determine the receiving sensitivities of UT transducers and AE sensors, a transmitter and a receiver are in direct contact, that is, the so-called face-to-face procedure is used. In lieu of pulse input, a transmitter is driven by a short segment of sinewave in generating reference vibration. This is a variation of Method 3 in ISO16063-11 [2]. Here, a wave packet is applied repeatedly on the transmitter, while measuring the receiver output. This sinewave method enabled an effective use of signal averaging technique to reduce the background of random noise contribution to the received output signals and allowed the determination of receiving sensitivities between 10 Hz and 1 MHz. Results are discussed in comparison to previously described receiving sensitivities from impulse methods and in regard to their frequency dependence. To determine sensor responses comprehensively, one needs a representative population and a total of 12 transducers and sensors are included in the present study: six for Group R (resonant), four for Group D (damping) and two for Group B (conical NIST type), respectively. Receiving sensitivity spectra are compared between the impulse and sinewave methods down to 1 kHz. Results are discussed regarding the level of agreement, effects of sensor loading, signal length and resonance. Resistive and capacitive loading effects are also accounted for, revealing open-circuit sensitivity behavior. The observed frequency response behavior will be discussed in terms of the damped harmonic oscillator (DHO) model [31,32], identifying three frequency response types, Types 0, 1 and 2. The type numbers mostly coincide with the exponent of power-law representation. Type 0 is for frequency independent spectra, Type 1 is linear frequency dependence and Type 2 is for power-law exponents of two or higher. Additionally, another region of Type 0 behavior is observed, and this arises from quasi-static piezoelectric response at low frequencies [28]. A section is added for possible improvements in sensitivity.

## 2. Experimental Procedures

Four models of UT transducers and eight models of AE sensors are examined in this study. These are Olympus V101 (0.5 MHz nominal center frequency, 25 mm diameter), V103 (1 MHz, 13 mm), V189 (0.5 MHz, 38 mm) and V192 (1 MHz, 38 mm) for the first group. For the AE sensor group: PAC R6a (60 kHz, 13 mm), R15a (150 kHz, 13 mm), Gesellschaft für Materialprüfung und Geophysik (GMuG, Bad Nauheim, Germany) (MA-Bls 40, 40–100, 30–200 and 20–400), a broadband KRN BB-PCP (1 mm) by KRN Services and an SE-1000H (by Score Atlanta, Houston, TX, a successor to DECI version without internal preamplifier). All twelve were evaluated for their receiving sensitivities, while V189 and V192 were also used as transmitters. Table 1 summarizes the transducers and sensors used. The nominal frequency of these transducers and some sensors will appear in parentheses with model name when available (except when it appears nearby).

**Figure 3:**
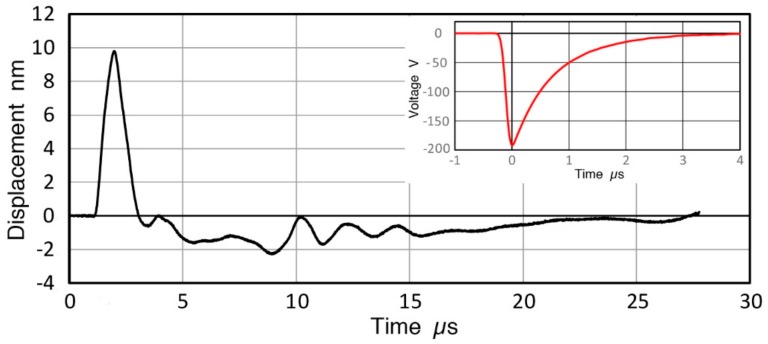
Pulse displacement waveform (in nm vs. µs) of V189 transducer with input voltage pulse waveform as an insert.

**Figure 4:**
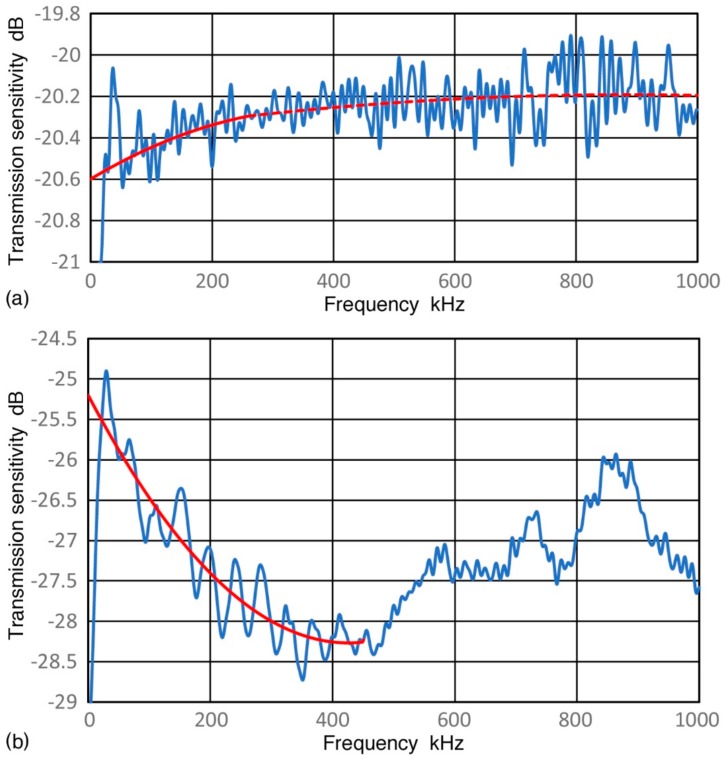
(**a**) Transmission displacement sensitivity of V189 (0.5) in dB in reference to 0 dB at 1 nm/V (blue curve). Curve-fit functions of Equation (1) plotted in red (<260 kHz) and Equation (2) in red dash (>260 kHz); (**b**) Transmission displacement sensitivity of V192 (1.0) in dB in reference to 0 dB at 1 nm/V (blue curve). Curve-fit function of Equation (3) up to 450 kHz plotted in red.

The pulse displacement waveform (in nm) of V189 transducer is shown in Figure 3 with an input voltage pulse waveform as an insert. Details of this test were described in [3,4]. A similar plot for V192 (1.0) can be found in [4]. With a spectral subtraction procedure, the transmission sensitivity of V189 (0.5) in displacement per V_in_ (in dB in reference to 0 dB at 1 nm/V) is given in Figure 4a. Figure 4b is the corresponding plots for V192 (1.0). The transmission sensitivities in velocity and in acceleration (in dB in reference to 1 (m/s)/V and to 1 (m/s^2^)/V) can be calculated by multiplying angular frequency 2πf (f is frequency in Hz) once and twice, respectively. The length unit was converted from nm to m. At frequencies below 1 MHz, V189 (0.5) has better transmission sensitivities in displacement than V192 (1.0) and is relatively flat with frequency. The difference is 6.0 dB at 100 kHz, increasing to 7.4 dB at 1 MHz. The displacement sensitivities contain numerous oscillations, especially for V189, and the transmission sensitivities, Tx, are represented by fitting second-order polynomial equations given below. The fitted curves are shown in red and red dash curves in Figure 4. For V189 (0.5), Equations (1) and (2) are used:

Tx = −2.225 × 10^−6^·f^2^ + 1.755 × 10^−3^·f − 20.60, 0 to 260 kHz (1)

Tx = −2.663 × 10^−7^·f^2^ + 4.709 × 10^−4^·f − 20.40, 260 to 1000 kHz. (2)

For V192 (1.0), curve-fitting was used only up to 450 kHz (Equation (3)) since the spectral shapes are too complex above.

Tx = 1.692 × 10^−5^·f^2^ − 1.440 × 10^−2^·f − 25.20 (3)

These transmission data are the basis for the present calibration method.

Direct comparison of sinewave and pulse methods was conducted using a laser interferometer (LH140, Thales, La Défense, France) for displacement measurements. This series was limited to 15, 20, 25, 30, 40 and 50 kHz to compare low frequency transmission sensitivities. Displacement transmission sensitivities measured by the two methods agreed to 0.55 ± 0.49 dB (average of six frequencies with the standard deviation).

A test was conducted to measure the level of vibration of sinewave-driven V189 transmitter. A two-second-long wave packet was applied to V189 (0.5), on which a G-Link-200 (Lord Sensing, Williston, VT, USA) accelerometer was pressed on using a jig with Vaseline couplant. Transmitter input voltage, V_in_, was between 55 and 134 V_rms_. Measured acceleration values were 0.0203, 0.0364 and 0.147 m/s^2^_rms_ at 200, 500 and 800 Hz, respectively. These were converted to displacement transmission sensitivities by dividing them with V_in_·(2πf)^2^, which resulted in −20.4, −23.5 and −22.5 dB in reference to 1 V/nm. The corresponding value of V189 Tx is −20.6 dB at these frequencies from the extrapolated Tx given by Equation (1). Thus, the differences are less than 3 dB between the ultrasonic impulse and accelerometer test results.

**Figure 5:**
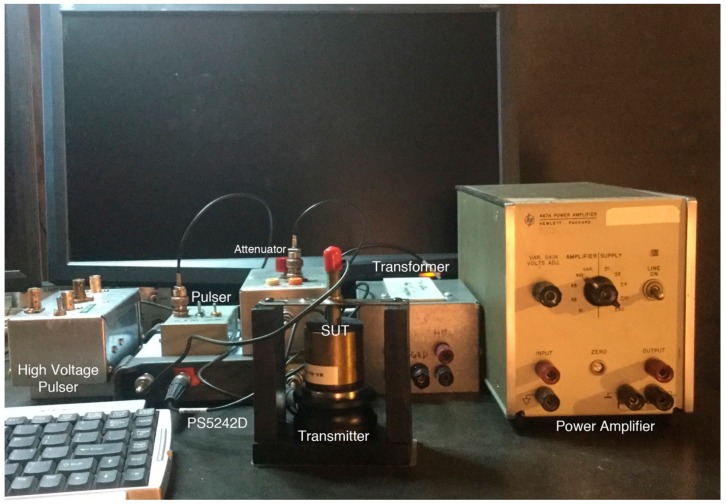
A photograph of the face-to-face test set-up.

The next step is to couple a sensor-under-test (SUT) to a transmitter, normally using V189 (0.5). V192 (1.0) was used when V189 was tested as a receiver. Vaseline was used as couplant between transmitter and receiver, which were pressed together with 25–35 N force, simultaneously monitoring receiver output to sinewave excitation. Figure 5 shows a photograph of face-to-face test set-up, with a jig holding a transmitter and an SUT along with other components as marked. A transformer and a high-voltage pulser are also shown, though unused in this set-up. After initial coupling, the set-up was left for at least one hour at 25 °C. When the receiver output reached a steady state, a wave packet, 15–45 cycles long sinewave at a test frequency, was applied repeatedly on the transmitter. While the number of cycles applied was varied by three times, the response was measured using 8 to 10 cycles avoiding transient-affected portions, especially at the start of the packet. The condition of coupling is more sensitive at lower frequencies below 20 kHz. In this range, ±1 dB was the limit of repeatability. Since this directly affects the precision of any face-to-face measurements, the same limit is present for all sensitivity data in this study. At higher frequencies, repeatability is better and a conservative value of ±0.5 dB can be used. The wave packet was generated by an arbitrary wave generator of PicoScope model PS5242D (Pico Technology, St Neots, UK). The output was fed to a power amplifier (HP467A, Hewlett-Packard, Palo Alto, CA, USA) through an attenuator for fine adjustment of amplifier output. This amplifier output of up to 5 V_rms_ was supplied to the transmitter and to one channel of PS5242D, while receiver output of a steady state portion (also in V_rms_) was measured in another channel of PS5242D, running with 14-bit resolution. In the series of tests using a laser interferometer, noted above, another power amplifier was used (HSA4011, NF Corp., Yokohama, Japan). In these tests, results are the root-mean-square (rms) voltages of the input to the transmitter and of the receiver output. A signal averaging function was active on both channels, which was especially effective in reducing background noise of the receiver signals. With a sensor (e.g., V189) connected to PS5242D, background noise level is 43.2 ± 1.3 µV_rms_ (with 16 ns sampling interval, 10 ms duration and 2-kHz low-pass filtering). By averaging 1000 times, it is reduced to 0.362 ± 0.008 µV_rms_. With a lower 200-Hz low-pass filtering, the averaged level is reduced further to 0.123 ± 0.005 µV_rms_. By using this method, the lowest signal amplitude measured was 3 µV_rms_ (at 10 Hz), while the minimum signal amplitude was typically 10–15 µV_rms_. Thus, signal-to-noise ratio higher than 30 dB was used in the present study. The signal averaging function was triggered by an external pulser with repeat frequency of 0.5 to 11 Hz. By dividing the output by input, converting it to dB scale and subtracting the transmission sensitivity at each frequency tested, the receiving sensitivity in dB (in reference to 0 dB at 1 V/nm) is determined. This approach allowed the determination of receiving sensitivities to 10–100 Hz on the low side and to 1 MHz on the high side. Typically, 40 to 60 frequency values were tested. For each decade, a sequence of 1, 1.2, 1.5, 2, 2.5, 3, 4, 5, 6, and 8 were usually used. More frequency values were added in some tests in order to compare with the results of impulse method. In these cases, resonant peak or antiresonance dip frequencies were chosen and frequency steps as fine as, for example, 0.1 kHz at 868 kHz, trying to reach the exact antiresonance frequency. However, the lowest values achieved were usually higher than those from the impulse data since the wave packets have broader spectral widths.

When receiving sensitivity is extremely low, one solution is to boost transmitter input. In place of HP467A, audio amplifiers of 30 to 80 W output were used in combination with a power transformer with 12.6 V and 600 V windings. Using these as the primary and secondary, a step-up ratio of ~25 was obtained up to 10 kHz. A peak transmitter input voltage to 2 kV was possible, but it was limited to 500 V in the present testing. The transformer was mostly used at frequencies below 100 Hz for the cases when the input impedance of the receiver was reduced to 10 kΩ or below to simulate a typical preamplifier input circuit. The normal input impedance used in this study was 1 MΩ of PS5242D. This difference of the input impedance is insignificant for most sensors at frequencies above 100 kHz. However, 30+ dB differences in receiving sensitivities can develop at frequencies below 100 Hz, where sensor’s capacitive impedance becomes high. This comparison will be a part of receiving sensitivity testing to be discussed below. Since higher transmitter voltages generated higher harmonics and distorted waveforms, most early high-voltage tests were repeated at normal low-voltage testing. The high-voltage set-up was used to obtain adequate output during comparison tests with an accelerometer.

The source of attenuation effects due to reduced input impedance is the increase in capacitive impedance of the piezoelectric sensors (Z_s_) at low frequencies. A widely used R15a (0.15) sensor has Z_s_ of 9–10 kΩ at 150 kHz, but this Z_s_ becomes about 15 MΩ at 100 Hz because Z_s_ = 1/2πfC_s_, where C_s_ is the sensor capacitance. For R15a, C_s_ = 110 pF, while C_s_ was 35 pF for a small KRN sensor and 2.42 nF for V189. When the input impedance of the measuring circuit is Z_in_, the ratio of a measured sensor voltage (V_in_) to a sensor generated voltage (V_s_) is given by

V_in_/V_s_ = Z_in_/(Z_in_ + Z_s_). (4)

**Figure 6:**
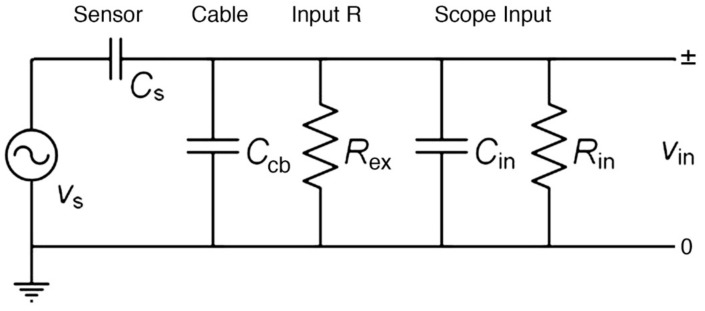
Equivalent circuit diagram of oscilloscope input. See text for symbols used.

For a typical input circuit used, the input impedance has four components as shown in Figure 6. These are cable capacitance, C_cb_, externally attached input resistance, R_ex_, and the input capacitance (C_in_) and resistance (R_in_) of the digital scope. Their impedance values are connected in parallel and Z_in_ is given by

1/Z_in_ = 1/R_ex_ + 1/R_in_ + 2πf(C_cb_ + C_in_). (5)

**Figure 7:**
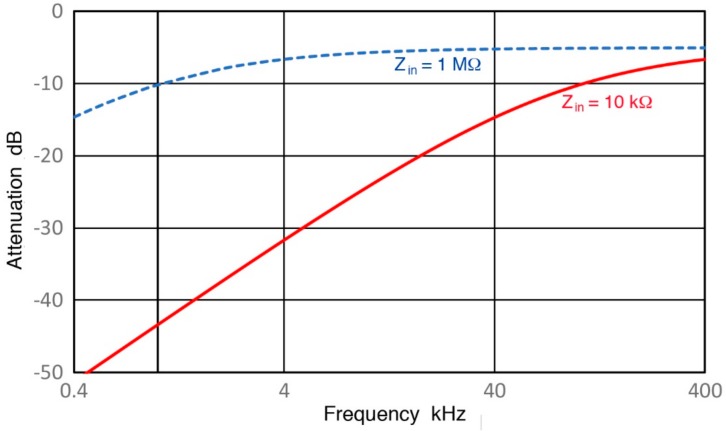
Attenuation caused by increasing sensor impedance with decreasing test frequency. Calculated results for R15a sensor with two input impedance of 1 MΩ (blue dash) and 10 kΩ red.

When an AE preamplifier is used, its Z_in_ is usually 10 kΩ and R_ex_ of 10 kΩ was used in our previous sensor studies [3,4]. In parts of this work, we removed R_ex_, leaving R_in_ of 1 MΩ and C_in_ of 14 pF. The values of C_cb_ are 60–100 pF for usual length of 60–100 cm in laboratory measurements. Figure 7 shows examples for R15a (0.15) sensor with or without an external resistor (R_ex_). With C_in_ of 110 pF and C_cb_ of 74 pF, an attenuation of 44 dB occurred at 1 kHz with R_ex_ of 10 kΩ, 34 dB more than the case without R_ex_ (or Z_in_ = 956 kΩ). In referring to the input impedance in the rest of this work, we use approximate values of Z_in_ = 1 MΩ, 10 kΩ and 380 Ω in lieu of the values of R_ex_.

By using Equation (4), V_s_ values can also be estimated from V_in_ measurements. These correspond to “open-circuit” sensor output voltages [1,33,34], that is, sensor output without loading effects. These are needed in comparing measurements with calculations from piezoelectric constants.

In connection to the above discussion on Z_in_, usual Z_in_ values for UT pulser-receivers are under 100 Ω while those for AE preamplifiers 10 to 20 kΩ. One may assume these are used unless other values are specifically mentioned. Some newer AE preamplifiers, such as Vallen AEP5 (Vallen Systeme, Icking, Germany) and NF SA240F5 (NF Corp., Yokohama, Japan), have Z_in_ of 1 MΩ. However, it is necessary to keep sensor cables shorter than 1 m to take advantage of such high input impedance. For AE applications that require long cable lengths, sensors with integrated preamplifiers need to be considered.

## 3. Results

### 3.1. V189 Transducer

**Figure 8:**
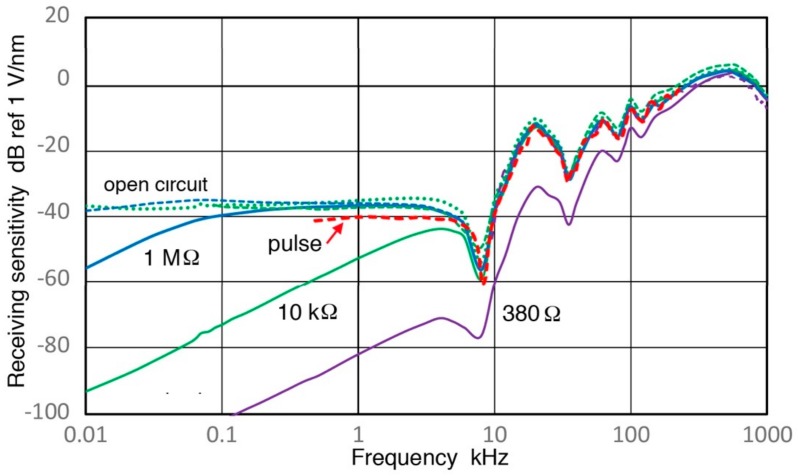
Receiving displacement sensitivity spectra are shown against log-frequency (in kHz) for V189 (0.5) transducer in dB in reference to 1 V/nm using three Z_in_ of 1 MΩ (blue), 10 kΩ (green) and 380 Ω (purple). Corresponding pulse sensitivity spectrum is in red dash curve and calculated open-circuit sensitivities are in blue dot (using data of Z_in_ of 1 MΩ) and in green dot (10 kΩ) curves. Third open-circuit sensitivity curve with 380 Ω is plotted but is invisible due to overlap.

This V189, belonging to Group D, is a large-size (38 mm in diameter) UT transducer with the nominal center frequency of 0.5 MHz. Receiving displacement sensitivities (Rx) of V189 were obtained using V192 (1 MHz, also 38 mm in diameter) as a transmitter using three Z_in_ values. Results for displacement sensitivities are shown in Figure 8. Here, solid curves in blue, green and purple are the displacement sensitivities for Z_in_ = 1 MΩ, 10 kΩ and 380 Ω, respectively. The values of Rx for two higher Z_in_ values differ less than 0.1 dB on average above 10 kHz. At lower frequencies below 5 kHz, the green Rx curve starts to decrease linearly with frequency, apparently exhibiting the so-called “velocity” response, where the velocity sensitivity is flat with frequency. This same trend also appears below 100 Hz for the blue Rx curve and for the purple Rx curve below 5 kHz. This apparent linear behavior is sometimes attributed to intrinsic sensor characteristics [23]. However, its dependence on Z_in_ values found here indicates that a different origin produces this observation. This linear dependence of Rx with frequency arises from increasing capacitive impedance of the sensor, or Z_s_, as the frequency decreases. As can be seen in Equation (4), the increased Z_in_ leads to essentially linear decrease in the scope input, V_in_, when we have Z_s_ >> Z_in_, thus producing Rx decrease in proportion to the frequency reduction.

At higher frequencies, an approximate linear frequency dependence can be also noted from 6 kHz to near the sensitivity peak at 500 kHz when sensitivity oscillations below 200 kHz are ignored. This represents the sensor behavior as impedance effects are small.

Because of frequency shift in the decreases in Rx, the addition of an external resistor produced a large drop in Rx. Figure 8 also shows large effects on Rx of the lowest Z_in_ value (380 Ω) below 200 kHz (purple curve). This was selected for a comparison with common geophones having coil resistance of 375–380 Ω as will be discussed later.

**Figure 9:**
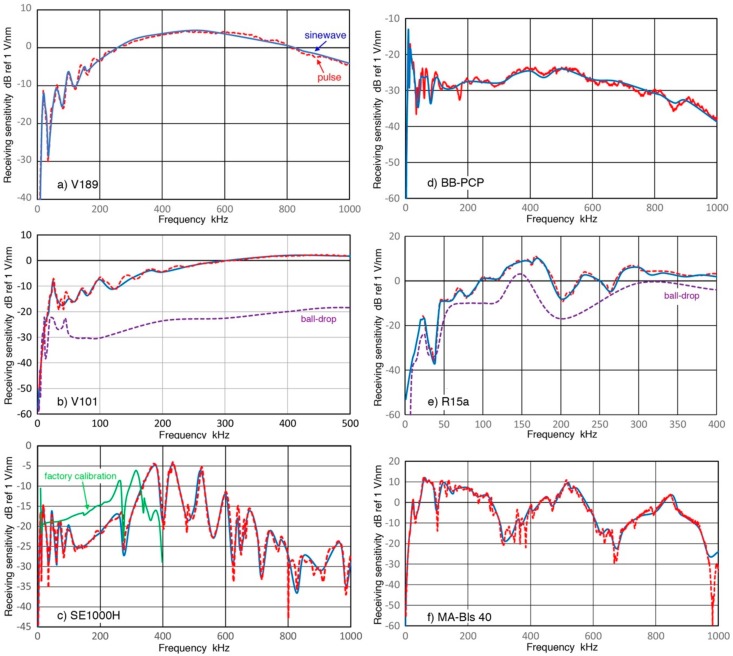
(**a**) Two sensitivity curves for V189 (Z_in_ = 1 MΩ) from Figure 8 are plotted in linear frequency scale in kHz. Receiving sensitivity spectra for sinewave (blue curve) and pulse (red dash curve) methods in dB scale as in Figure 8; (**b**) Same as in (**a**) for V101 from Figure 11; (**c**) Same as in (**a**) for SE1000H from Figure 12; (**d**) Same as in (**a**) for KRN BB-PCP from Figure 13; (**e**) Same as in (**a**) for R15a from Figure 14; (**f**) Same as in (**a**) for MA-Bls-40 from Figure 15.

The Rx values with Z_in_ = 1 MΩ also match the Rx data of the impulse test (also used Z_in_ = 1 MΩ, shown by red dash curve in Figure 8) within 1.49 ± 1.11 dB on average for 1 kHz to 1 MHz. Below the 8 kHz dip, the pulse data is a few dB lower (3.3 dB at 1 kHz). This agreement in Rx at >50 kHz can be shown more clearly in linear frequency plot, given in Figure 9a. The same color codes are used. Note that this comparison was made between the receiving sensitivities that used Z_in_ = 1 MΩ. The observed agreement between the present sinewave method and the pulse excitation approach [3,4] indicates that both techniques can be utilized for receiving sensitivity determination. The level of the standard deviation (1.11 dB) appears to reflect variations in the coupling conditions and to represent the limitation of the face-to-face arrangement. A part of the standard deviation was also from that of transmission sensitivities as noted in Section 2. In this study, the duration of the received signals in face-to-face tests increased to 250–950 µs in order to recover low-frequency signals buried in noise through the use of signal averaging. The impulse data for V189 (Figure 8) used 600 µs length. Although the energy of the trailing signals is quite low, the trailing signals following the main pulse contribute to oscillations observed below 100 kHz. A signal duration of at least 100 µs is needed to capture resonance related spectral oscillations. Additional tests were conducted and will be discussed in a later section.

When the attenuation effects from high sensor impedance are corrected using Equation (4), receiving sensitivities based on the values of open-circuit sensor output are obtained and these are given as dotted curves in Figure 8. The same color codes are used. The three curves agreed well, giving an average difference of less than 1 dB. This finding indicates that the method used can determine the open-circuit sensor sensitivities. These curves coincided with the high impedance blue Rx curve above 4 kHz, while becoming essentially constant at −36.0 ± 1.0 dB for 0.01–4 kHz, which corresponds to 15 mV/nm. This frequency independent response seems to imply a quasi-static piezoelectric response [26,27,28,29]. This is unexpected for a high-frequency UT transducer, but indicates the basic piezoelectric phenomenon. Such behavior is normally hidden since a high-impedance input circuit is required to observe it. The present work appears to be the first to report this phenomenon in high frequency UT/AE detectors.

**Figure 10:**
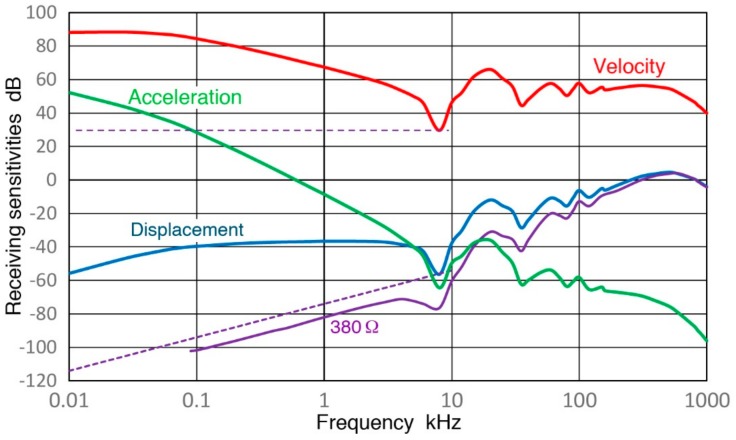
Sinewave displacement sensitivity curve for V189 (Z_in_ = 1 MΩ: blue curve) is converted to velocity (red) and acceleration (green) sensitivity curves, showing a flat velocity region at >50 kHz. This is for comparison with a typical geophone, whose velocity (upper purple dash line) and displacement (lower purple dash) responses are given along with Rx for 380 Ω purple curve).

Figure 10 gives plots of the high-Z_in_ sensor sensitivities in terms of displacement (blue curve), velocity (red) and acceleration (green). The sensitivity conversion used the division with 2πf and (2πf)^2^, as discussed earlier. The velocity sensitivity (red) curve does indicate a nearly flat region at higher frequencies (>50 kHz). Also shown in Figure 10 are two purple dashed lines. The upper thin line corresponds to the velocity sensitivity (~32 V/m/s) of a typical geophone with a coil resistance of 380 Ω (Model GS-11D, Geospace Technologies, Houston, TX, USA), while the lower thick line represents its displacement Rx. The latter is about 10 dB higher than the V189 Rx with R_ex_ of 380 Ω, implying that V189 (0.5) has comparable Rx with a slightly higher R_ex_ value (1.1 kΩ). This higher R_ex_ also provides a comparable velocity Rx for V189 to that of GX-11D.

### 3.2. V101 Transducer

**Figure 11:**
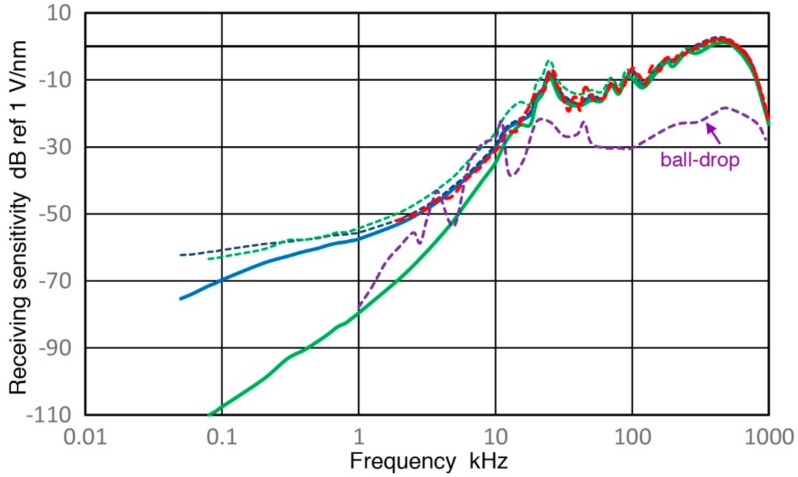
Receiving displacement sensitivity spectra are shown against log-frequency (in kHz) for V101 (0.5) transducer in dB in reference to 1 V/nm using two Z_in_ of 1 MΩ (blue) and 10 kΩ (green) and 380 Ω (purple). Corresponding pulse sensitivity spectrum is in red dash curve and calculated open-circuit sensitivities are in blue dot (using data of Z_in_ of 1 MΩ) and in green dot (10 kΩ) curves. A spectrum obtained by a ball-drop method is plotted for comparison in purple dash.

This is another UT transducer (Group D) with the nominal center frequency of 0.5 MHz. Receiving displacement sensitivities (Rx) of V101 were obtained using V189 (0.5) as before and results for Rx are shown in Figure 11. Here, solid curves in blue and green are the displacement sensitivities for Z_in_ = 1 MΩ and 10 kΩ. The values of Rx for these Z_in_ values differ less than 1.3 dB above 10 kHz. At lower frequencies below 3 kHz, the green curve starts to decrease with f^1.5^, exhibiting a steeper response than the apparent velocity response. At frequencies higher than 5 kHz, the slopes become steeper to 2 to 3, the latter for the 10 kΩ impedance. At still higher frequencies, past a minor resonance peak at 25 kHz, the frequency dependence decreases to that of the velocity response between 40 and 500 kHz, excluding resonance-induced spectral oscillations. This velocity response was also found in the case of V189. After correcting effects of increasing capacitive impedance, the open-circuit Rx (shown in blue and green dotted curves) continued their decreases below 1 kHz with f^0.33^ dependence. These two curves match well at high (>100 kHz) and low (<1 kHz) frequencies, but are a few dB apart in the mid-frequency range of 1 to 100 kHz.

The Rx values also matched the Rx data of the impulse test (both Z_in_ of 1 MΩ), shown by red dash curves in Figure 9b and Figure 11 within 0.35 ± 0.75 dB on average above 10 kHz. As before, a linear frequency plot, Figure 9b shows the matching behavior clearly, indicating both techniques can obtain Rx values satisfactorily.

Both figures for V101 (0.5) include purple dash curves. These are Rx values obtained by a ball-drop method [23]. The general trend of the purple curves roughly matches the present Rx data with Z_in_ of 10 kΩ. This behavior is indicative of V101 possessing low frequency sensitivities as demonstrated here and in [23]. Above 20 kHz; however, the ball-drop Rx values become 10 to 30 dB lower than the clustered curves of this study. This attenuation comes from the use of hot melt glue for sensor attachment as reported in [23]. It also appears that a preamplifier was used in [23], giving a rough match between the green and purple curves < 10 kHz.

### 3.3. SE1000H Sensor

**Figure 12:**
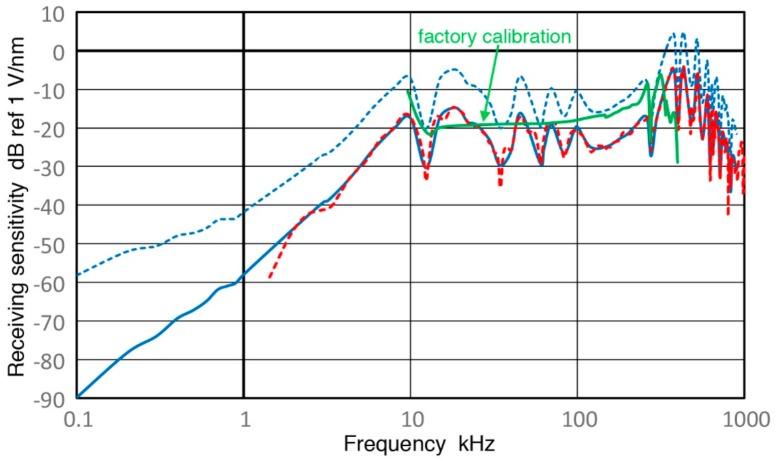
Receiving displacement sensitivity spectra (both Z_in_ of 1 MΩ) are shown against log-frequency (in kHz) for SE1000H sensor in dB in reference to 1 V/nm. One is with sinewave method (blue). The other is corresponding pulse sensitivity spectrum, given in red dash curve. Calculated open-circuit sensitivity is in blue dot (using sinewave data of Z_in_ of 1 MΩ) curve. Factory calibration curve is plotted for comparison in the green curve.

This AE sensor belongs to Group B and has a 1-mm diameter sensing area with a sensor housing size of general use AE sensors. This was developed at DECI about twenty years ago as noted in Section 1. The manufacturer’s sensitivity data showed it to be possibly a high-performance sensor. Receiving displacement sensitivities (Rx) of SE1000H were obtained using V189 with Z_in_ = 1 MΩ and spectral results for Rx are shown in Figure 12. Here, blue and red curves are Rx using the sinewave method and impulse method, respectively. These two curves agree well from 2 kHz to 1 MHz with average difference of 0.26 ± 1.37 dB. This agreement can also be confirmed in a linear frequency plot of Figure 9c with the same color code as in Figure 12. Another curve plotted in Figure 9c is the open-circuit sensitivity of sinewave Rx, given as a blue dashed curve. Because of its small capacitance of 45 pF, impedance correction is large even above 100 kHz, where such a correction tends to become negligible.

In Figure 9c and Figure 12, manufacturer’s sensitivity calibration is shown by a green curve. Evidently, the green curve is better behaved than our Rx results. However, their data sheet offers no information as to the nature of the calibration, such as the direction of wave motion, surface or normal incidence, the basis of calibration standards, etc. They must be utilizing a quite different calibration standard. Details of their method are unknown, except that they follow the ASTM E-976 guide for sensor performance evaluation [35]. Unfortunately, ASTM E-976 is overly broad and offers no clue in regard to any specific calibration approach and face-to-face method is not included.

It is interesting to note that this sensor has the highest sensitivity peak beyond their specifications, which are cut off at 400 kHz. As Figure 9c shows, three highest peaks occur at 432, 370 and 525 kHz. The peak sensitivity values are approximately −5 dB in reference to 1 V/nm. However, this Rx value is misleadingly low since its sensing area is much smaller than most other sensors, typically with a 10-mm diameter sensing area or larger. As shown in [3], the sensitivity for normal incidence waves increases proportionately with the sensing area. Thus, Rx for small aperture sensors, as these are usually known, can be 20 to 40 dB higher, when Rx is measured per unit sensing area. This implies that this sensor has a superior sensitivity when a test object is small or its contact area is limited.

### 3.4. KRN BB-PCP Sensor

**Figure 13:**
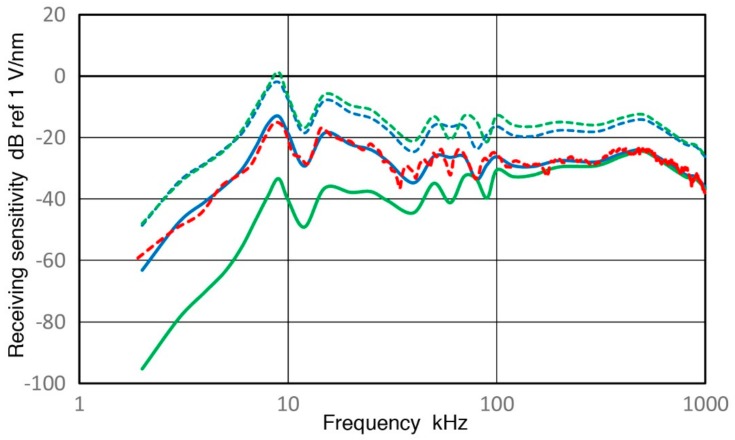
Receiving displacement sensitivity spectra (Z_in_ = 1 MΩ and 10 kΩ) are shown (in blue and green) against log-frequency (in kHz) for KRN BB-PCP sensor in dB in reference to 1 V/nm. The corresponding pulse sensitivity spectrum is given in red dash curve. Calculated open-circuit sensitivity is in blue and green dot curves (using sinewave data of Z_in_ of 1 MΩ and 10 kΩ).

This KRN BB-PCP sensor also belongs to Group B and is another sensor with a small sensing area. It is also with a 1-mm diameter aperture and was previously evaluated for its receiving sensitivities for both normal incidence waves [3] and for guided wave sensing [36,37]. Here, Rx of BB-PCP sensor were obtained using V189 (0.5) with Z_in_ = 1 MΩ and 10 kΩ. The spectral results for Rx are shown in Figure 13 with blue (1 MΩ) and green (10 kΩ) curves representing the sinewave method and red dash curve for the impulse method, respectively. Two open-circuit sensitivities are given with blue and green dotted curves, as before. These two curves are a few dB apart in the mid-frequency range of 20 to 500 kHz, but match well at other frequencies. As with the three sensors above, the Rx obtained by the sinewave method and the impulse method agree with each other between 3 kHz and 1 MHz. The average spectral difference was 0.05 ± 1.72 dB, indicating an overall excellent match with a scatter. The level of the standard deviation was comparable to the case of V189 (0.5) in Section 3.1. Figure 9d shows the corresponding spectral plots for Rx in linear frequency scale. The observed Rx spectra for the KRN sensor are closer to a flat response than SE1000H above, which has a series of resonance peaks that are nearly 20 dB higher. These two sensors serve complementary roles in their uses in various AE testing applications.

### 3.5. R15a Sensor

**Figure 14:**
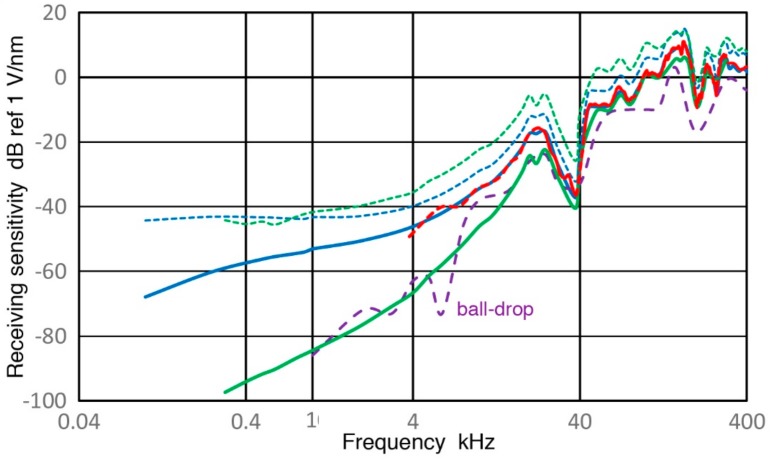
Receiving displacement sensitivity spectra are shown against log-frequency (in kHz) for R15a (0.15) sensor in dB in reference to 1 V/nm using two Z_in_ of 1 MΩ (blue) and 10 kΩ (green). Corresponding pulse sensitivity spectrum is shown in the red dash curve and calculated open-circuit sensitivities are in the blue dot (using data of Z_in_ of 1 MΩ) and green dot (10 kΩ) curves. A spectrum obtained by a ball-drop method is plotted for comparison in purple dash.

This belongs to Group R and is a general-use AE sensor with the nominal center frequency of 150 kHz. Rx values of R15a were obtained using V189 (0.5) as before and results for Rx are shown in Figure 9e and Figure 14. The values of Rx for two Z_in_ values differ about 1 dB above 40 kHz. Both curves have a series of peaks and dips and the highest resonance peak was found at 170 kHz. An overall trend between 40 and 400 kHz is the 1.5-power dependence on frequency, or f^1.5^. At lower frequencies below 25 kHz, the green curve starts to decrease steeply, followed by less steep slope below 4 kHz, tending toward the apparent velocity response below 1 kHz. The slope of the high-Z_in_ Rx curve in blue is always lower. After correcting effects of increasing capacitive impedance, the open-circuit Rx (shown in blue and green dotted curves) decreases in parallel to the blue Rx curve to 4 kHz, but reaches a plateau below 2 kHz (at 43.4 ± 0.4 dB). As in the case for V189, this frequency-independent Rx values suggest the presence of the quasi-static response of a piezoelectric sensing element. These two curves match well at high (>100 kHz) and low (<1 kHz) frequencies, but are ~5 dB apart in the mid-frequency range of 3 to 200 kHz.

The values of Rx obtained by the sinewave method (blue) and the impulse method (red) agree with each other between 4 kHz and 400 kHz. The average spectral difference was 0.23 ± 0.95 dB, indicating a good overall match with a low scatter. The level of the standard deviation was slightly higher than 0.75 dB for V101 sensor in Section 3.2. This good agreement is better shown in linear frequency plot, given in Figure 9e. This figure also shows clearly large attenuation of 5–15 dB of the ball-drop Rx (purple dash curves) above 100 kHz [23]. Below 100 kHz, the ball-drop curve roughly follows the present Rx data with Z_in_ of 10 kΩ indicating preamplifier use and is close to the clustered Rx curves above 30 kHz. This is indicative of approximate agreement between the present methods and the ball-drop approach below 100 kHz.

While the present testing of R15a may appear strange, since this study is aimed at low frequency sensor behavior, some results are surprising. For example, let us compare at 1 kHz between Rx values of R15a and a geophone, GX-11D, discussed in Section 3.1. Here, we have Rx (R15a, 1 MΩ) = −53 dB and Rx (GX-11D, 380 Ω) = −73 dB. When a short cable can be used, 20 dB sensitivity advantage can be gained with R15a. This turns to 10 dB loss when 10-kΩ Z_in_ has to be used with a preamplifier. Thus, common AE sensors should not be dismissed for low frequency applications just because their peak sensitivities occur at much higher frequencies.

### 3.6. GMuG MA-Bls 40 Sensor

**Figure 15:**
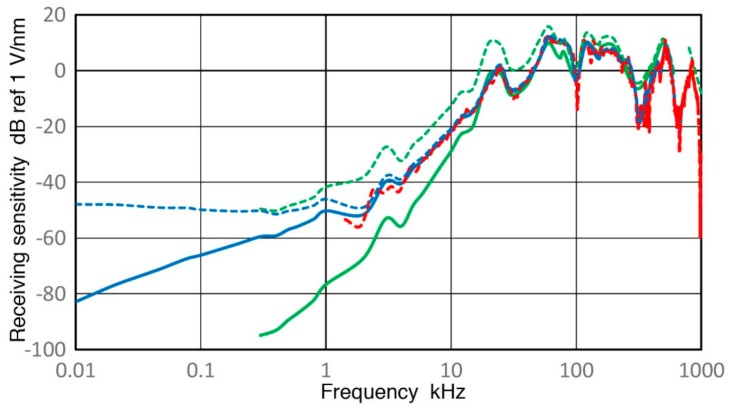
Receiving displacement sensitivity spectra are shown against log-frequency (in kHz) for MA-bls 40 sensor in dB in reference to 1 V/nm using two Z_in_ of 1 MΩ (blue) and 10 kΩ (green). Corresponding pulse sensitivity spectrum is in red dash curve and calculated open-circuit sensitivities are in blue dot (using data of Z_in_ of 1 MΩ) and in green dot (10 kΩ) curves.

This also belongs to Group R and is an AE sensor developed at GMuG for use in underground AE monitoring. It has a relatively large size of 40 mm diameter, with high sensitivity at frequency ranges of 50–270 kHz, 500–550 kHz and 800–870 kHz, as shown in Figure 9f and Figure 15. Rx values of MA-Bls 40 were obtained using V189 (0.5) as before. The values of Rx for these Z_in_ values mostly trace each other above 15 kHz except at 60–100 and 300–500 kHz. Both curves have a series of peaks and dips and the highest Rx peaks were found at 62 and 515 kHz. An overall trend for the high Z_in_ data is f^0.8^ dependence below 1 kHz, followed by its slope reaching near two for 2–70 kHz and finally between 70 and 1 MHz is nearly flat peak response with frequency. With a lower Z_in_, the green curve starts to rise with f^2^ below 2 kHz, and the slope gets larger becoming f^4^ dependence between 10 and 20 kHz. After correcting effects of increasing capacitive impedance, the open-circuit Rx (blue and green dotted) curves start as a near plateau below 2 kHz and then increase as f^2^ in parallel to the high Z_in_ (blue dot) curve to 70 kHz. This is similar to the Rx behavior of V189. The low-frequency plateau of open-circuit Rx values suggest the presence of the quasi-static response of a piezoelectric sensing element. The low Z_in_ (green dot) curve is 8–10 dB higher than the blue dot curve over 1 to 50 kHz. This behavior was also found in most other cases examined above.

The Rx values obtained by the sinewave and impulse methods agree with each other between 2 kHz and 970 kHz. The average spectral difference was 0.37 ± 1.48 dB, indicating a good overall match with a scatter. The level of the standard deviation was comparable to those of V101(0.5), SE1000H and KRN sensors. This agreement is more clearly seen in the linear frequency plot, given in Figure 9f.

### 3.7. Other Sensors Examined

Six other sensors have been similarly tested. These include two in Group D, V192 (1.0), V103 (1.0), and four in Group R, R6a (0.06), MA-Bls 40–100, MA-Bls 30–200 and MA-Bls 20–400. Their results are comparable to those in the same grouping presented above: V192 and V103 to V189 (0.5) and V101 (0.5), R6a to R15a (0.15) and three MA-Bls sensors to MA-Bls 40. Consequently, their main data in the form of two figures each are given in Appendix A with short comments.

## 4. Discussion

### 4.1. Sinewave and Impulse Methods

For twelve transducers/sensors, receiving displacement sensitivities obtained by two calibration methods, sinewave excitation and impulse excitation methods, are compared. The sensors include those presented in Appendix A with only a brief description. The average spectral differences and their standard deviation are summarized in Table 2. These average values are below 1 dB (except V189) and the standard deviation values are less than 2 dB. These results indicate that the two calibration methods yield identical receiving sensitivities. The graphical results given in Appendix A for the remaining six sensors are consistent with the present conclusion. All these sensors showed good agreement between the sinewave (blue) and impulse (red dash) curves.

The confirmation of the equivalency of sinewave-based and pulse-based methods has now answered a long-standing question, namely, the suitability of using long-duration signal analysis methods like swept-frequency spectrum analysis for the evaluation of UT/AE sensors for pulse detection. Such swept-frequency methods have long been used widely, but now we can point to this equality when a query is received.

### 4.2. Pulse Length Effects

**Figure 16:**
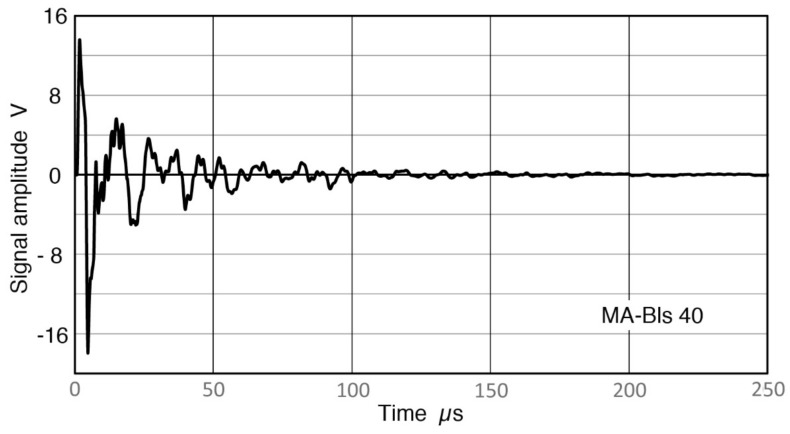
Receiver output waveform to 250 µs. V189 transmitter and MA-Bls 40 receiver. Signals near noise level beyond 150 µs can be recovered by signal averaging to 585 µs.

**Figure 17:**
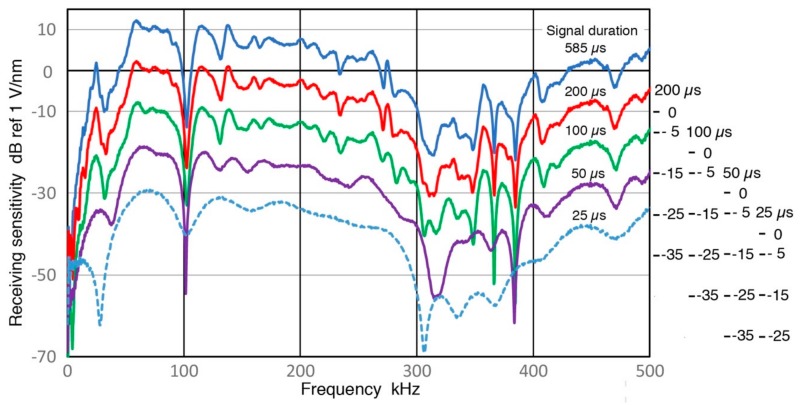
Receiving sensitivity spectra of MA-Bls 40 using different signal length of the waveform in Figure 16. Signal lengths used were 585, 200, 100, 50 and 25 µs. The results are in blue, red, green, purple and blue dot curves in decreasing order of signal length. Each spectrum is shifted by 10 dB vertically and shifted scales shown at right.

**Figure 18:**
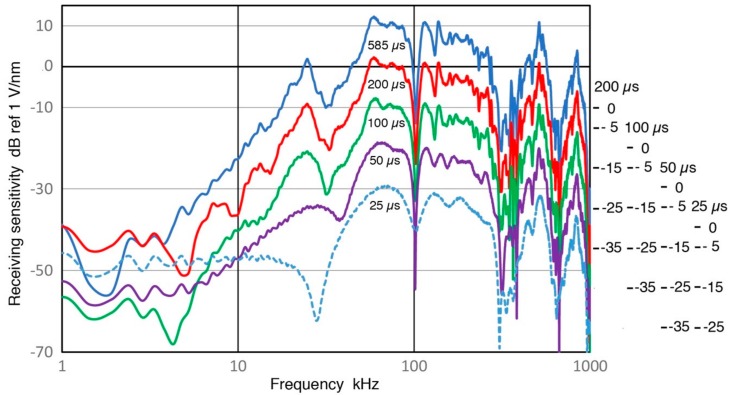
The same receiving sensitivity spectra of MA-Bls 40 from Figure 17, but are shown with log-frequency scale to expand the low frequency region. Each spectrum is shifted by 10 dB vertically and shifted scales shown at right.

It is necessary to increase the length of a pulse signal in order to properly evaluate spectral contents of the pulse at lower frequencies, as noted in Section 3.1. A sensor output from MA-Bls 40 with Z_in_ of 1 MΩ is shown in Figure 16. Results of Section 3.6 were obtained from this signal, taking the spectral data from 585-µs segment. In this figure, signal amplitude apparently reaches the baseline level beyond 150 µs. However, it does contain valid low-frequency receiver signals to 585 µs, which can be viewed by scale expansion. For this evaluation, signal length was reduced from 585 to 200, 100, 50 and 25 µs and receiving sensitivities of the sensor were recalculated. From the waveforms, the signal energy contents beyond 100 µs appear to be small and inconsequential. Even the portion between 50 and 100 µs may be ignored if one is only interested in the signal energy. The recalculated Rx data are given in Figure 17. The top curve is for the longest (585 µs) signal, while the bottom curve is for 25-µs long signal, with each spectrum staggered by 10 dB for visibility. The differences between 585 µs (blue) and 200 µs (red) spectra are indeed slight, except significant changes are visible when the data is plotted in log-frequency scale, shown in Figure 18. As the signal length is reduced further, spectral features are increasingly lost, especially at frequencies below 50 kHz. Even for 50 kHz or higher, 50-µs duration is needed to retain the main features visible in the long signal spectrum. Obviously, these changes are the consequence of signal length reduction, which is equivalent to the imposition of a square-shaped time-domain window. From these figures, it appears we a need signal duration of ~500 µs when spectral fidelity of 5 kHz is needed, ~200 µs for 15 kHz and above, and ~100 µs for 30 kHz and above. This approximate rule roughly corresponds to three times the inverse signal duration. Please note that such long duration signals must be acquired using a stable signal averaging procedure so that noise is minimized.

### 4.3. Open-Circuit Sensor Sensitivity

**Figure 19:**
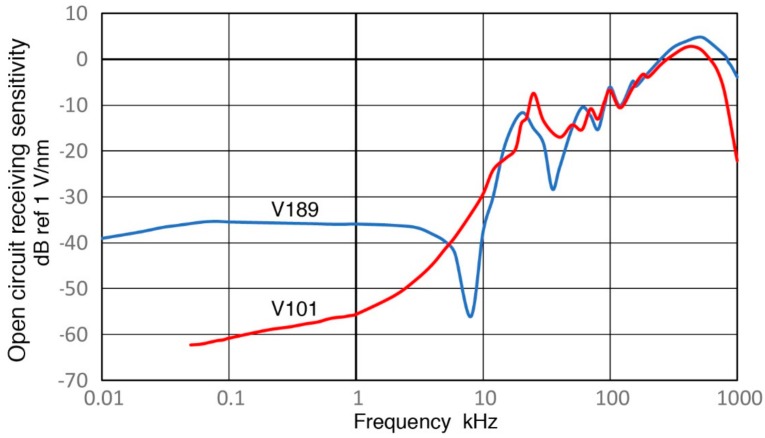
A comparison of two open-circuit receiving sensitivity spectra for V189 (0.5: blue) and V101 (0.5: red). Log-frequency scale.

**Figure 20:**
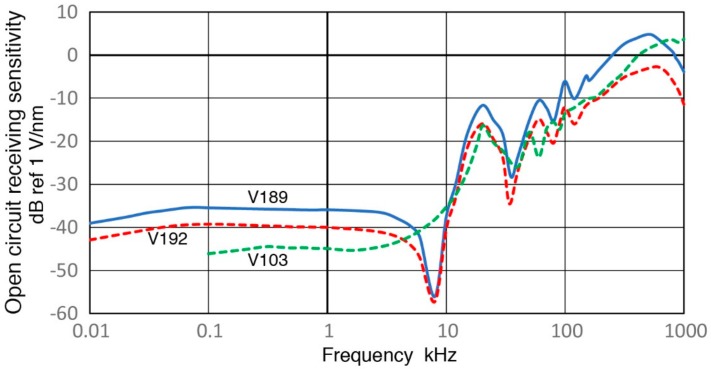
Three open-circuit receiving sensitivity spectra for V189 (0.5: blue), V192 (1.0: red dash) and V103 (1.0: green dash) are compared. Log-frequency scale.

**Figure 21:**
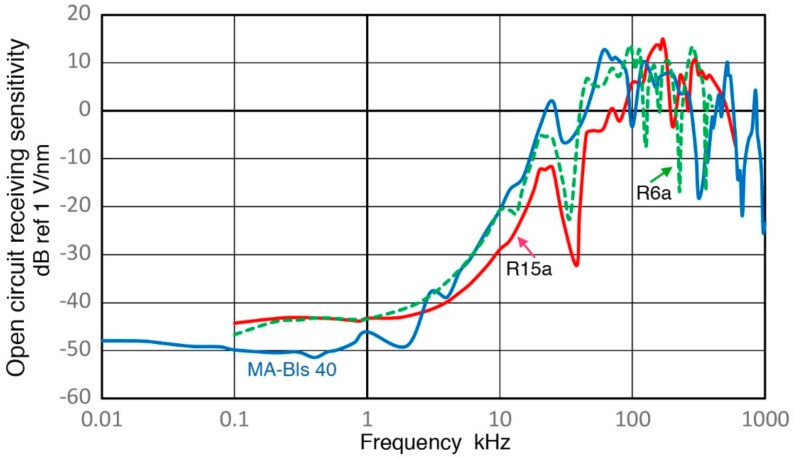
Three open-circuit receiving sensitivity spectra for R6a (0.06: green dash), R15a (0.15: red) and MA-Bls 40 (blue) are compared. Log-frequency scale.

**Figure 22:**
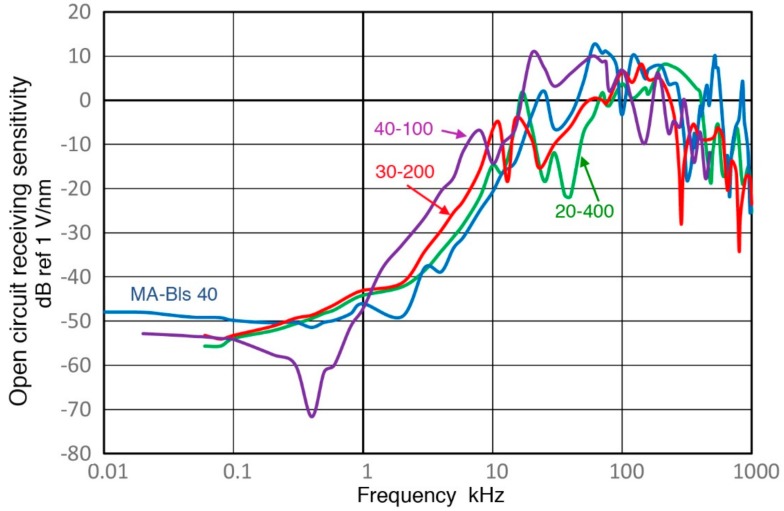
Four open-circuit receiving sensitivity spectra for GMuG sensors are compared. Log-frequency scale. MA-Bls 40 (blue); MA-Bls 40–100 (purple); MA-Bls 30–200 (red); MA-20–400 (green).

**Figure 23:**
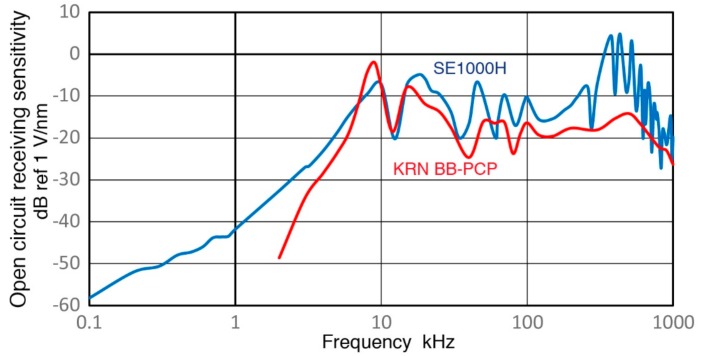
A comparison of two open-circuit receiving sensitivity spectra for SE1000H (blue) and KRN BB-PCP (red). Log-frequency scale.

Observed receiving sensitivity spectra shown in Section 3 have been corrected for the attenuation effects due to increasing sensor impedance (Z_s_) as the test frequency is reduced. This correction produced corresponding open-circuit sensitivity spectra. Figure 19, Figure 20, Figure 21, Figure 22 and Figure 23 replot the open-circuit sensitivity spectra thus obtained (designated as Rx^∞^), grouping similar sensors together. Only the results for high-impedance (Z_in_ of 1 MΩ) tests are used for clarity. Figure 19 shows Rx^∞^ for two UT transducers, V189 (0.5) and V101 (0.5). Both exhibited approximately linear frequency responses or the slope of unity over 40 to 500 kHz. Below a minor peak at 19 or 25 kHz, Rx^∞^decreases sharply with the slope of 5 or 2.3 for V189 or V101. As the frequency goes down to 3 kHz for V189 or 1 kHz for V101, the frequency dependence becomes nearly absent (V189) or low (V101). Two other UT sensor results are compared with that of V189 in Figure 20. This comparison includes V192 (1.0) and V103 (1.0) from Appendix A. Their behaviors parallel the V189 curve (blue), except that a dip at 8 kHz was absent (green dash curve). Below 3 kHz, Rx^∞^ was nearly unchanged with frequency in all three. In Figure 21, Rx^∞^ spectra of three resonant AE sensors, R6a (0.06, in green dash curve, data from Appendix A), R15a (0.15, red) and MA-Bls 40 (blue) are compared. In all three, Rx^∞^ decreases sharply below the highest sensitivity peaks (at 100, 170 and 60 kHz, respectively) with the slope of two (ignoring large dips at 30–40 kHz). Below 2–3 kHz, the frequency dependence again diminishes as in Figure 20. Figure 22 shows Rx^∞^ behaviors for all four GMuG sensors with MA-Bls 40 (in blue curve), MA-Bls 40–100 (in purple curve), MA-Bls 30–200 (in red curve) and MA-Bls 20–400 (in green curve). The data for the last three sensors is from Appendix. While their Rx^∞^ responses have many oscillations above 20 kHz, f^2^-dependence below the lowest resonance peak was always present. Low or diminished frequency dependence was also found in all four sensors at low frequencies (except for a large dip for MA-Bls 40–100 at 400 Hz). Figure 23 shows Rx^∞^ behaviors for SE1000H and BB-PCP. In both of the small aperture sensors, Rx^∞^ shows the sensitivity levels between 5 and −20 dB in reference to 1 V/nm above the lowest resonance peak at ~10 kHz. In contrast, Rx^∞^ decreases rapidly with f^2^ for SE1000H or f^3.5^ for BB-PCP below the 10-kHz peak. A low frequency dependence region was not detected for these two sensors as the sensitivity decreased rapidly.

### 4.4. Modelling Dynamic Sensor Behavior

The observed frequency dependence of Rx^∞^ can best be understood in terms of the damped harmonic oscillator (DHO) model, yielding the governing equation of sensor dynamics, developed for inertial seismometers [31]. A common vibration model of a mass supported by spring in parallel to a damper is considered to represent a seismometer and it has been diverted to piezoelectric AE sensors [32]. With x as displacement input, y as sensor motion or displacement, ω_o_ as angular resonance frequency and η as damping constant, we have

(6) $$-\frac{d^{2}x}{{\mathrm{dt}}^{2}}=\frac{d^{2}y}{{\mathrm{dt}}^{2}}+2{\eta \omega }_{o}\frac{\mathrm{dy}}{\mathrm{dt}}+\omega_{o}^{2}y$$

and its solution for y in terms of ω/ω_o_ = f/f_o_ with angular frequency ω and resonance frequency f_o_,

|y(f/f_o_)| = (f/f_o_)^2^/{[1 – (f/f_o_)^2^] + 4η (f/f_o_)^2^}^0.5^. (7)

**Figure 24:**
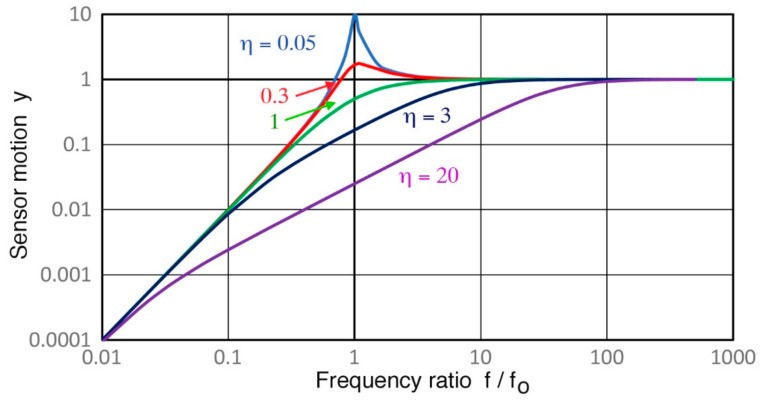
Sensor motion (y) vs. frequency ratio, f/f_o_, representing the solution of the sensor dynamics equation or DHO model. See Equation (7).

The solutions using η values of 0.05, 0.3, 1, 3 and 10 are plotted in Figure 24. The top two curves represent under-damped conditions with resonant behavior. The third curve for unity η is called critical damping, while the two lowest curves (η > 1) represent over-damping. In all cases, the slope is zero or constant amplitude for large values of f/f_o_ or f/f_o_ > 1. This is referred to as Type 0. On the other side at f/f_o_ < 1, the slope is two where acceleration is constant. In over-damped (η > 1) cases, the unity slope or velocity response is found centering at f/f_o_ ≈ 1 between the low and high frequency ranges.

This over-damped case corresponds to the unity slope region in V189 (0.5) and V101 (0.5), both highly damped, found in Figure 19 over 40 to 500 kHz and in Figure 20 for V192 (1.0) and V103 (1.0). This behavior is designated as Type 1. Next, Type 2 behavior is related to under-damping, for which a resonance peak exists and the slope is high on either side of the peak as shown by the blue curve for η = 0.05. Type 2 behavior is exhibited in four damped UT transducers (Figure 19 and Figure 20), in six resonance sensors (Figure 21 and Figure 22) and in two small aperture sensors (Figure 23). In all these units, steep slopes of 2 to 5 are observed below the resonance peaks and the observed behavior matches that of under-damped cases (η < 1). Specific frequency ranges were described in Section 3 for each transducer/sensor. Often, Type 2 behavior is found in over-damped UT transducers, since, even in these transducers, lower frequency resonances due to radial modes are present. These low-frequency resonances from radial modes appear to be undamped as backing materials are designed for suppressing thickness resonances.

**Figure 25:**
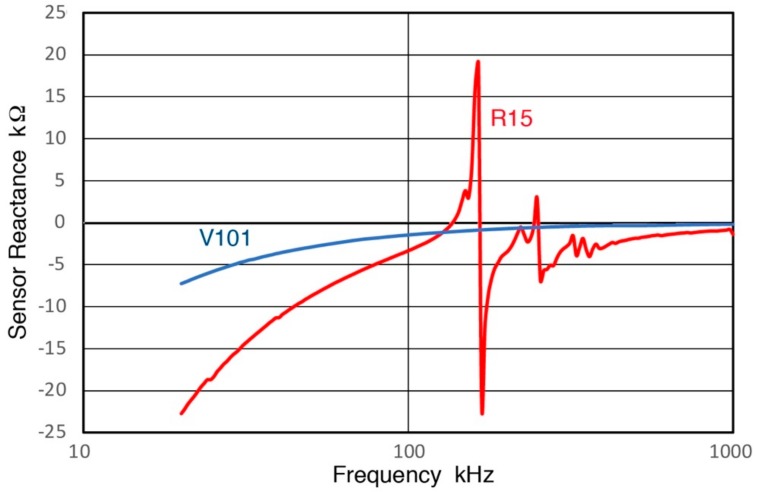
Reactive components of Z_s_ are plotted against frequency for V101 (0.5) and R15 (0.15).

A puzzling feature on Figure 20 and Figure 22 is a dip in the spectra at ~8 kHz for V189 and V192 and at 0.4 kHz for MA-Bls 40–100. This apparent antiresonance dip has no corresponding resonance peak at a lower frequency. The sensor impedance Z_s_ at low frequencies is determined by the capacitance, so Z_s_ starts at a large negative value, as shown in Figure 25 for V101 (0.5) and R15 (0.15). Here, the reactive components of Z_s_ are plotted against frequency, while |Z_s_| vs. frequency curves for V101 and R15 (comparable to R15a, Group R) were given in Figure 17 of reference [3]. When the reactance reaches zero, |Z_s_| is at a minimum and exhibits a resonance (at 137 kHz for R15). This is followed by a maximum, giving an antiresonance at 164 kHz. For V101, a Group D sensor, both the reactance and |Z_s_| do not reach zero. The impedance spectra, however, are poor indicators of resonance behavior. As Figure 11 and Figure 14 show, more than several prominent peaks exist in the Rx spectra. This indicates that radial resonances have no effect on |Z_s_| spectra. The |Z_s_| spectra also did not correlate with transmission spectra [3]. Similar findings are discussed in [38,39]. Thus, it appears that these dips are not from antiresonance, but are due to the extension of the high-slope (Type 2) region below a resonance peak, before transitioning to the quasi-static (Type 0) region.

Another unresolved issue is the presence of lower frequency resonances below the fundamental radial and thickness resonance frequencies. This was examined by Ohtsu and Ono [40] using finite element analysis method for piezoelectric elements. They found that circular bending and radial-compression modes are dominant for a free disk and produced several sub-harmonic resonances. However, coupling to a backing mass makes analysis too complicated. Their mesh size was still large due to memory limitation in 1983 and better analysis is needed. Andrade et al. [39] also observed sub-harmonic resonances through finite element analysis of composite piezoelectric elements, while conventional analysis only showed fundamental resonance. This method may resolve the issue found here, but some works ignore mode coupling effects [38].

In the small aperture sensors, a sensing element is backed by a mass to provide displacement response, as originally developed at the NIST [8]. In terms of Figure 24, this corresponds to the Type 0 region of f/f_o_ > 1, where the slope is zero. Unlike the NIST sensor’s 250-g backing, however, SE1000H (43 g total weight) and BB-PCP (17 g) have much smaller mass sizes. Thus, resonance effects cannot be damped adequately at lower frequencies. This appears to produce the accelerometer-like Type 2 responses found below 10 kHz in SE1000H and BB-PCP.

**Figure 26:**
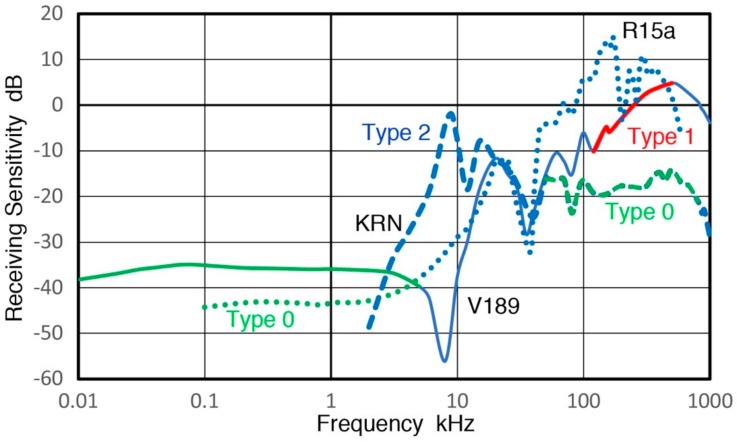
Three types of frequency dependence illustrated for V189 (Group D), R15a (Group R) and KRN (Group B).

From the above examination of observed frequency dependence of R_x_^∞^, it can be concluded that the DSO model accounts for the displacement response (Type 0) with large mass, the velocity response of damped UT transducers over a broad frequency range below the main resonance (Type 1) and steep slopes of 2 to 5 found below local resonance peaks in all transducers/sensors studied (Type 2). These three types and quasi-static behavior are illustrated in Figure 26. Type 0 is given in green curves at <5 kHz for V189 (solid) and R15a (dotted) and for the mass-loaded KRN sensor (dashed) at 30–800 kHz. Type 1 is in red for V189 over 120–500 kHz, while Type 2 behavior is shown in blue curves for all three sensors in the mid-frequency region.

### 4.5. Quasi-Static Piezoelectric Response

The damped harmonic oscillator model discussed above predicts a flat displacement sensitivity zone above the resonance frequency (higher if damped). However, the motion always diminishes as the frequency is decreased below the resonance with a steep slope of more than two regardless of damping condition. Thus, the frequency independent R_x_^∞^ behavior discovered in many of 12 sensors at low frequencies as shown in Figure 19, Figure 20, Figure 21, Figure 22 and Figure 23 has to be explained by another phenomenon. Earlier (in Section 3.1), quasi-static piezoelectric response was suggested as a possible source. This subject has been treated in the literature in recent years [26,27,28,29] and is of particular importance in actuator applications.

**Figure 27:**
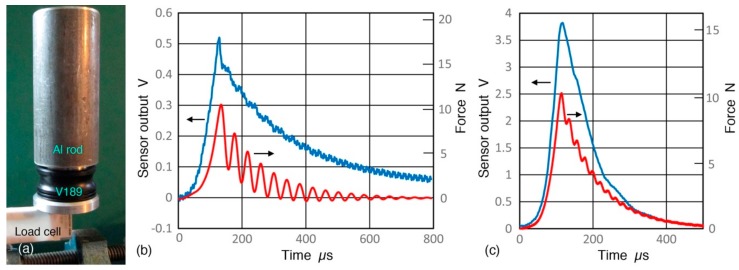
(**a**) Unloading test set-up; (**b**) Waveforms of sensor output by rapid unloading of 10.7 N using 100 MΩ input impedance (blue curve) and force (red curve). 100 Hz low-pass filter was inserted. V189 transducer; (**c**) Same as (**b**) with PZT-5A disk.

**Figure 28:**
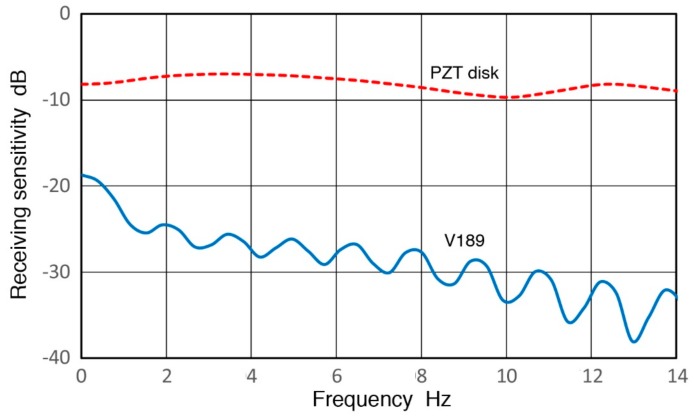
Receiving sensitivities of V189 (blue) and PZT disk (red dash) vs. frequency.

This possibility was examined by placing an aluminum rod (1.099 kg mass, 61 mm diameter, 135 mm length) on V189 (0.5) and rapidly lifting it, while its output was measured on PicoScope through a 1/100-x probe with 100 MΩ input impedance (see Figure 27a). Applied force was also measured using a strain gage load cell with a conditioner). An exemplar of output waveforms is shown in Figure 27b. Average peak height was 487 ± 17 mV. This shows that even a simple quasi-static test induces relatively large output from a UT transducer. Thus, the quasi-static response hypothesis appears to be worth exploring further. In the absence of design details of V189, the output value is calculated in terms of output voltage per unit force, resulting in 45.5 mV/N or −26.8 dB in reference to 1 V/N. Frequency responses of the output voltage and force taking Fourier transform of the data in Figure 27b also produced the output-voltage/force response as a function of frequency, as shown in Figure 28 (blue curve). This frequency spectrum is modulated and changes about 10 dB over 0 to 10 Hz. An average over 4 ± 2 Hz is −26.9 dB in good agreement with the result from peak values. This frequency range approximately correlates to the rise time of the output of 0.12 ms (or ~4 Hz). The observed spectral oscillations were absent when a load cell was not used, indicating the source to be from a combination of the load cell and sensor mass.

Another series of tests with a PZT-5A disk (18 mm diameter, 5.35 mm thickness) were also conducted. Disk output from 10.7 N load removal on this PZT disk was 3.97 ± 0.06 V (using Z_in_ = 100 MΩ). Sensor output and force are shown in Figure 27c) corresponding to a peak output voltage per unit force of 371 mV/N or −8.6 dB. This value is 18 dB higher than that of V189 (0.5) transducer. Its frequency response is given in Figure 28 (red dash curve) and an average over 6 ± 3 Hz is −7.7 dB, matching the peak value data within 1 dB. The spectrum is flatter than that for V189 to 14 Hz shown, varying ±2 dB. The rise time of the disk output was 0.08 ms, implying a 50% higher frequency of 6 Hz. The difference observed between a PZT element and V189 is expected since the latter is heavily damped to obtain broad bandwidth.

For this PZT disk, the displacement can be calculated from its Young’s modulus of 50 GPa as 4.21 nm. The receiving sensitivity or output voltage per unit displacement is 0.948 V/nm. This sensitivity value is −0.45 dB or 7.1 dB lower than a piezoelectric constant, h_33_ = 2.15 V/nm (6.65 dB), which is the displacement coefficient for a thin disk [30,41]. A part of the difference comes from the impedance effect even with the use of Z_in_ = 100 MΩ. Since the signal half-width was 0.08 s, its center frequency is estimated as 6 Hz, which results in impedance correction of 9.6 dB as the disk capacitance is 544 pF. The observed sensitivity agrees to h_33_ within 2.5 dB using the peak value data (or 1.4 dB with the spectral method), since the difference is within expected variation of piezoelectric coefficients. Thus, the present test method is confirmed to be valid. Please note that the theoretical sensitivity or the h_33_ value of PZT-5A is of the same order of the peak resonance sensitivities of most sensors considered in the present study. For example, R6a sensor (0.06) has peak sensitivity of 4–6 dB, R15a has 10 dB and V103 (1.0) has 2 dB in reference to 1 V/nm.

In the case of V189 transducer, the same calculation cannot be used as no elastic stiffness data is available. When a PZT-5A disk of 38 mm diameter and 4 mm thickness (for getting 500 kHz resonance) is subjected to the same 10.7-N unloading, the product of h_33_ and displacement in the thickness direction provides the output voltage [30,41]. The displacement is found using the Young’s modulus of 50 GPa as 0.71 nm. If this is assumed as V189 displacement, its observed output sensitivity becomes 0.685 V/nm (−3.2 dB). With 1.3-dB impedance correction, it is 0.90 V/nm (−0.92 dB) or 7.6 dB less than the h_33_ value of PZT-5A. However, this lower sensitivity value is still much larger than R_x_^∞^ of V189 (0.5) of −38.2 dB at 10 Hz or 12.2 mV/nm from Figure 8. In the above, the output voltage per unit force was reduced by 18 dB in V189 compared to a bare PZT disk, which was attributed to damping [30,34]. Additional discrepancy can be expected by a lower elastic stiffness of V189 sensing element, increasing the displacement and decreasing sensitivity per unit displacement. The use of composite piezoelectric element can reduce the stiffness [39] and this may be a possible cause for the observed difference. Different test and analysis methods of single frequency sinewave packets vs. impulse or of the use of peak values vs. frequency spectral methods can be ruled out considering the results presented above.

Despite the need to further clarify the origin of lower sensitivities of damped transducers at low frequencies below 1 kHz, as noted above, it is evident that quasi-static displacement sensitivity is found in an undamped PZT disk, the value of which is comparable to the inherent PZT sensitivity (h_33_). In damped transducers, the sensitivity level is lower and, for V189 (0.5), the observed level was approximately 40 dB below h_33_. The observed frequency independent displacement response can be attributed to the piezoelectric element proper.

### 4.6. Enhancing Low-Frequency Response

The observed behavior of large transducers, V189 and V192, suggests that Rx improvements at low frequencies can be obtained via quasi-static piezoelectric effect. The sensitivity levels are governed by the piezoelectric constant, h_33_. However, this effect requires high input impedance, which can be realized by using an internal preamplifier. Another obstacle to achieving low frequency sensitivity is the presence of resonances, below which a sharp sensitivity loss occurs. When one examines observed resonance behavior below 50 kHz in Section 4.3, some peak frequencies correspond to fractions of expected fundamental radial resonance. In damped Group D transducers like V189 and V192 (see Figure 20), the lowest resonance was near 20 kHz and the quasi-static region commenced at 4 kHz. These transducers are of 38-mm diameter and we should expect the radial resonance at ~50 kHz. Here, it is necessary to assume that a disk-shaped sensing element of nominal sensor diameter for the lack of sensor construction detail. In another example, consider R15a from Group R. Its predecessors (e.g., PAC R15) had 12.7-mm diameter and 6.4-mm thickness, giving the radial resonance at 157 kHz and thickness resonance at 282 kHz using PZT-5A parameters. These two frequencies approximately corresponded to the highest and next highest Rx peaks for R15a in Figure 14. However, there are four more peaks below the main peak at 170 kHz, the last one being at 20 kHz. In this case, Type 2 behavior lasted from 20 kHz to 4 kHz and quasi-static effect was found only below 4 kHz. Small aperture sensors (Group B) had the lowest resonance at 8–9 kHz and exhibited no quasi-static region. Thus, we need to keep the frequency of the lowest resonance above 15–20 kHz to utilize the quasi-static effects for low-frequency Rx enhancement. It is likely that the sub-harmonic resonances are due to mode coupling as revealed by the finite element analysis [40]. However, the origin of the low-frequency resonance needs further studies as discussed in Section 4.4 [38,39,40].

Another approach appears possible by following the NIST conical sensor design principle. This utilizes Type 0 behavior with a large mass backing, thus reducing the resonance frequency. The size of such sensors has to become larger than traditional AE sensors, however. For increasing size of sensing element, it may be worth considering the use of cylindrical elements. Some AE sensors have used small cylinders over the years and this may also work at low frequencies by adding backing mass.

While the methods for improving low-frequency Rx are still vague without clear understanding of the physical basis, e.g., sub-harmonic radial resonance, it is best to avoid Type 2 region of rapid Rx decrease as seen in the small aperture sensors (see Figure 23). These small sensors are not suitable for low frequency applications. The discussions and suggestions in this section are still preliminary. Further efforts are needed to develop high-sensitivity AE sensors for examining large structures using low frequency waves. Needs for such capability are in high demand globally.

## 5. Conclusions

Receiving sensitivities of UT and AE transducers/sensors are evaluated using sinewave packet excitation method and compared to the corresponding data from pulse excitation method with a particular emphasis on low frequency behavior below 20 kHz. Both methods rely on the determination of transmitter displacement characteristics using a laser interferometric method.

1. Receiving displacement sensitivities obtained by two calibration methods, sinewave excitation and impulse excitation methods, are compared and a good agreement is found. The average spectral differences are below 1 dB and the standard deviation values are less than 2 dB. These results indicate that the two calibration methods yield identical receiving sensitivities.
2. As the test frequency is reduced, effects of attenuation increase substantially due to increasing sensor impedance and require correction in order to evaluate the inherent sensitivity of a sensor. This is known as open-circuit sensitivity, which differs often by more than 20 dB from customary use of preamplifiers having input impedance of about 10 kΩ.
3. Input impedance effects lead to apparent velocity response below 100 kHz for typical AE sensors. This arises from a voltage divider circuit formed by the sensor capacitance and input impedance and gives linear frequency response.
4. Sensors with large mass backing show flat displacement response like NIST sensor (Type 0). Damped broadband sensors and UT transducers exhibit inherent flat velocity response below their main resonance frequency with linear frequency dependence (Type 1). In sensors with an under-damped resonance peak, a steep sensitivity decrease occurs below the resonance showing frequency dependence of f^2^~f^5^ (Type 2). Such behaviors originate from sensor characteristics that can best be described by the damped harmonic oscillator model.
5. At low frequencies, typically below 1 kHz, receiving sensitivity exhibits frequency independent behavior (Type 0). Seven of 12 sensors showed this flat region, while three more appear to approach the flat region. This appears to originate from the quasi-static piezoelectric response of the sensing elements in these sensors.
6. In using the impulse method for low frequency region, it is necessary to include often discarded tail parts of waveform, recovering low level signals with signal averaging. With signal duration of ~500 µs, spectral fidelity is obtained for 5 kHz and above, ~200 µs for 15 kHz and above and ~100 µs for 30 kHz and above.

## Figures and Tables

**Table 1:** UT transducers and AE sensors.

Manufacturer	Model	Nominal Frequency (MHz)	Element Diameter (mm)	Note
Olympus	V189	0.5	38	Main
Olympus	V192	1	38	Appx
Olympus	V101	0.5	25	Main
Olympus	V103	1	13	Appx
PAC	R6a	0.06	13	Appx
PAC	R15a	0.15	13	Main
Score Atlanta	SE1000H	Broadband	1	Main
KRN Services	KRN BB-PCP	Broadband	1	Main
GMuG	MA-Bls 40	0.13, 0.07	40 *	Main
GMuG	MA-Bls 40–100	100 ^†^	40 *	Appx
GMuG	MA-Bls 30–200	200 ^†^	30 *	Appx
GMuG	MA-Bls 20–400	400 ^†^	20 *	Appx

* Outside diameter; ^†^ Upper limit frequency; Main: presented in the main text; Appx: presented in Appendix A.

**Table 2:** Average spectral differences between two calibration methods.

Sensor	Ave. Difference (dB)	Standard Deviation (dB)	Low Cut-Off (kHz) *
V189 (0.5)	1.49	1.11	1
V192 (1.0)	0.64	1.66	1
V101 (0.5)	0.25	0.79	2
V103 (1.0)	0.81	1.18	2
SE1000H	0.26	1.37	2
KRN BB-PCP	0.05	1.72	2
R6a (0.06)	0.40	1.65	10
R15a (0.15)	0.48	0.95	4
MA-Bls 40	0.37	1.48	2
MA-Bls 40–100 ^†^	0.19 (0.75)	1.28 (3.16)	2
MA-Bls 30–200	0.04	0.63	2
MA-Bls 20–400	0.09	1.13	2

* Averages are for frequency range between low cut-off and high frequency limit of 1 MHz (400 kHz for R6a and R15a). ^†^ Two dips at 655 and 868 kHz excluded. Values in parentheses without exclusion.

**Figure A1:**
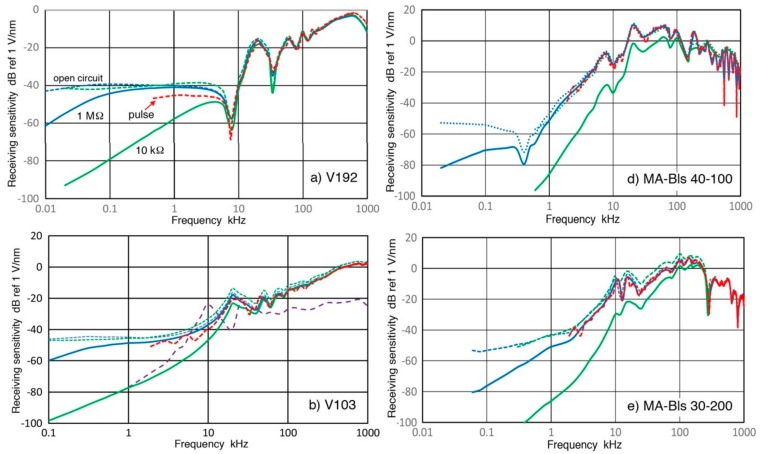

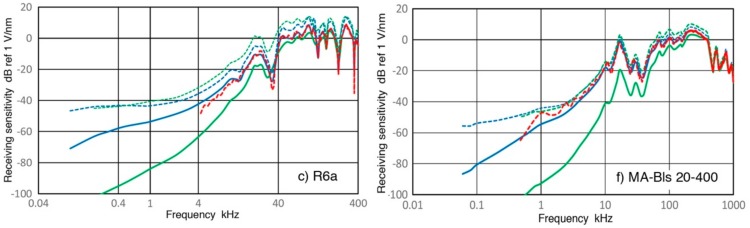
(**a**) Receiving displacement sensitivity spectra are shown against log-frequency (in kHz) for V192 (1.0) transducer in dB in reference to 1 V/nm using two Z_in_ of 1 MΩ (blue) and 10 kΩ (green). Corresponding pulse sensitivity spectrum is in red dash curve and calculated open-circuit sensitivities are in blue dot (using data of Z_in_ of 1 MΩ) and in green dot (10 kΩ) curves; (**b**) Same for V103 (1.0); (**c**) Same for R6a (0,06); (**d**) Same for MA-Bls 40–100; (**e**) Same for MA-Bls 30–200; (**f**) Same for MA-Bls 20–400.

**Figure A2:**
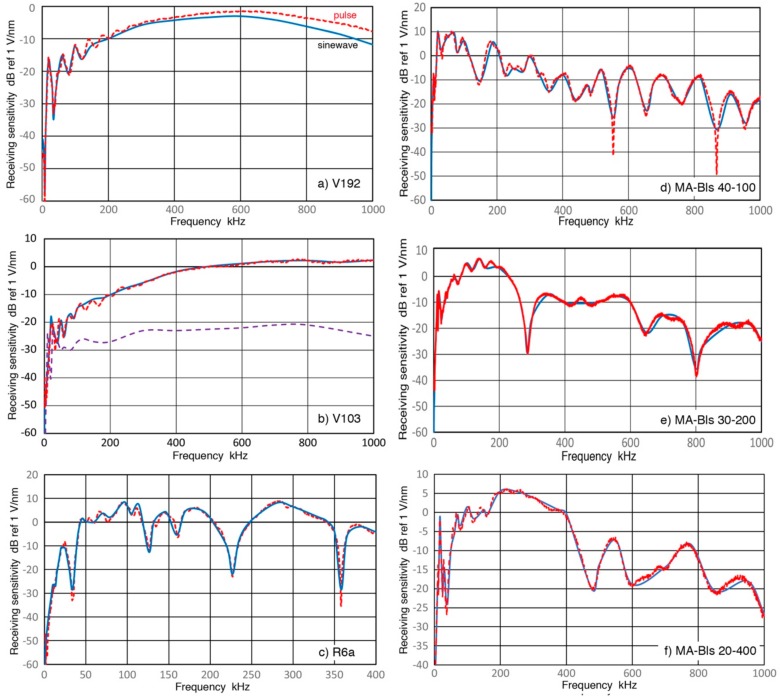
(**a**) Two sensitivity curves for V192 (Z_in_ = 1 MΩ) from Figure A1a are plotted in linear frequency scale in kHz. Receiving sensitivity spectra for sinewave (blue curve) and pulse (red dash curve) methods in dB scale as in Figure A1a; (**b**) Same for V103 (1.0); (**c**) Same for R6a (0.06); (**d**) Same for MA-Bls 40–100; (**e**) Same for MA-Bls 30–200; (**f**) Same for MA-Bls 20–400.

## Appendix A

Test results for two more UT transducers and four more AE sensors are presented below. These are given without elaboration, since transducers and sensors with similar construction are discussed above. Their counterparts are noted in the following.

A1. V192 transducer (Group D): V192 (1.0) is a 38-mm diameter UT transducer similar to V189 (0.5). See Figure A1a and Figure A2a for receiving sensitivities (Rx). Its Rx^∞^ was compared to V189 and V103 in Figure 19. Its Rx values were several dB lower than V189. Matching between sinewave and pulse methods was good at 0.64 ± 1.66 dB for 1 kHz to 1 MHz frequency range. Differences were large below 4 kHz (e.g., 4.4 dB at 1 kHz) and above 600 kHz.

A2. V103 transducer (Group D): V103 (1.0) is a 13-mm diameter UT transducer and its response resembles those of V101 (0.5) and V192 (1.0), but with better high frequency response. See Figure A1b and Figure A2b for receiving sensitivities (Rx). Its Rx^∞^ was compared to V189 and V192 in Figure 20. For this transducer, Vallen [42] recently determined peak velocity sensitivities at 15–20 kHz for three units using a laser vibrometer and an Olympus V104 (2.25) transmitter. His results were 0, −2.0 and −2.4 dB in reference to 1 V/m/s. When the data from Figure A1b and Figure A2b are converted to velocity sensitivities, our data yield 0.0 dB for the sinewave and −2.5 dB for the pulse method, respectively. Excellent agreement is obtained, providing another validation between independent studies.

A3. R6a sensor (Group R): R6a (0.06) is a 13-mm diameter AE sensor and its response resembles those of R15a and MA-Bls 40. See Figure A1c, Figure A2c and Figure 21. Its peak responses appear at 95 and 280 kHz rather than its nominal resonance of 60 kHz. As noted earlier, an AE sensor with 78-kHz resonance peak showed 1.2 mV/m/s^2^ to 10 kHz [14], which corresponds to displacement sensitivity of −88 dB at 1 kHz. This is close to R6a value of −85 dB. It appears both have similar design.

A4. MA-Bls 40–100 sensor (Group R): This has 40-mm diameter casing and is for underground use. See Figure A1d and Figure A2d. Figure 22 shows that it has the best response over 1 to 50 kHz in comparison to three other GMuG sensors.

A5. MA-Bls 30–200 sensor (Group R): This has 30-mm diameter casing and is for underground use. See Figure A1e and Figure A2e and its Rx^∞^ was compared to other GMuG sensors in Figure 22. Its response is nearly 10 dB lower than 40–100 sensors at 2–10 kHz, but is comparable to other GMuG sensors generally.

A6. MA-Bls 20–400 sensor (Group R): This has 20-mm diameter casing and is for underground use. See Figure A1f and Figure A2f and its Rx^∞^ was compared to other GMuG sensors in Figure 22.
